# Multifunctional Fe_3_O_4_@ZIF-8 Nanoparticles with Antibiosis and Osteogenesis for Treatment of Jaw Osteomyelitis

**DOI:** 10.3390/pharmaceutics18030359

**Published:** 2026-03-13

**Authors:** Heng Li, Zhiyue Zhang, Yu Wang, Ting Mou, Jiaqi Tian, Chong Huang, Lu Zhao, Zeyang Ge, Dandan Wang, Chenlu Li, Jihong Wang, Yanzhen Zheng, Lei Tian, Chunlin Zong

**Affiliations:** 1 State Key Laboratory of Oral & Maxillofacial Reconstruction and Regeneration, National Clinical Research Center for Oral Diseases, Shaanxi Clinical Research Center for Oral Diseases, Department of Oral and Maxillofacial Surgery, School of Stomatology, The Fourth Military Medical University, Xi’an 710032, China; 17798815711@163.com (H.L.); zhangzy126830@163.com (Z.Z.); mtmuting@126.com (T.M.); huangchongson@163.com (C.H.); zhaolujiayou0513@163.com (L.Z.); 18792863533@163.com (Z.G.); 17791823751@163.com (D.W.); lcl17309283208@outlook.com (C.L.); wangjh0129@163.com (J.W.); 2 Frontier Institute of Science and Technology, Interdisciplinary Research Center of Frontier Science and Technology, Xi’an Key Laboratory of Electronic Devices and Material Chemistry, School of Future Technology, Xi’an Jiaotong University, Xi’an 710054, China; 4121128016@stu.xjtu.edu.cn (Y.W.); zheng.yanzhen@xjtu.edu.cn (Y.Z.); 3 School of Stomatology, Jiamusi University, 258 Xuefu Street, Xiangyang District, Jiamusi 154007, China; 4 Xi’an Gaoxin No. 1 High School, No. 78 Xifengfu Road, Yanta District, Xi’an 710119, China; 13891949360@163.com

**Keywords:** jaw osteomyelitis, Fe_3_O_4_@ZIF-8 nanoparticles, antibacterial activity, bone regeneration, heat shock response

## Abstract

**Background/Objectives**: Jaw osteomyelitis (OM) is a refractory purulent inflammation caused by bacterial infection, characterized by persistent infection, excessive bone resorption, and resultant bone defects. Currently, mainstream therapies for jaw OM struggle to eradicate persistent infections, avoid antibiotic resistance, and repair infected bone defects, posing a critical challenge in clinical practice. **Methods**: Herein, the Fe_3_O_4_@ZIF-8 core–shell nanoparticles (NPs) platform designed for jaw OM treatment consisted of Fe_3_O_4_ as the core and zeolitic imidazolate framework-8 (ZIF-8) as the shell. **Results**: The core–shell platform not only integrated the pH-responsive degradation capability of ZIF-8 but also retained the superparamagnetism of Fe_3_O_4_ NPs. In the acidic, infectious microenvironment, Fe_3_O_4_@ZIF-8 NPs underwent continuous degradation, releasing Zn^2+^, thereby conferring potent antibacterial activity. The specific antibacterial mechanism of the nanoparticles lies in the fact that high concentrations of Zn^2+^ directly disrupted bacterial cell membranes and inhibited the bacterial heat shock response. This dysregulates bacterial proteostasis, rendering the bacteria more sensitive to external adverse stresses, ultimately leading to bacterial death. With ZIF-8 framework degradation, the encapsulated Fe_3_O_4_ NPs were released. Under static magnetic field (SMF) synergy, Fe_3_O_4_ NPs collaborated with Zn^2+^ to promote bone regeneration and repair infected bone defects in jaw OM lesions. **Conclusions**: As a multifunctional core–shell platform, Fe_3_O_4_@ZIF-8 NPs meet the dual clinical needs of antibiosis and osteogenesis, offering a promising translational strategy for jaw OM therapy.

## 1. Introduction

Jaw osteomyelitis (OM) is a purulent inflammation of the jawbone involving cortical bone, cancellous bone, and bone marrow [1]. Epidemiologically, the global annual incidence of acute jaw OM is 5–10 cases per 100,000 population, with the mandible being the most frequently affected site, accounting for 60–85% of all cases [2]. Jaw OM is characterized by recurrent and persistent infections, excessive bone resorption, and resultant bone defects, and is often accompanied by pus, sequestrum, and fistula formation, severely impairing oral function and quality of life [3]. Current standard treatment for jaw OM follows the core principle of “infection control before defect repair” [4]. Firstly, infected tissue and sequestrum are removed by debridement, with systemic antibiotics used to control infection. Subsequently, bone grafting or bone repair materials are used to reconstruct jaw defects. However, this approach presents multiple challenges in clinical practice. On one hand, prolonged and improper antibiotic use increases the risk of bacterial resistance, microbial imbalance, and systemic toxicity [5]. On the other hand, incomplete infection control and delayed repair of infected bone defects contribute to a persistently high recurrence rate [6]. Furthermore, current clinical options for treating bone defects, including autogenous bone grafting [7], allograft bone transplantation [8], bioceramics [9], titanium alloy scaffolds [10], and artificial bone graft materials [11], are generally lacking natural antibacterial properties. When applied to jaw OM-related regions at a high risk of infection, they still require long-term antibiotic support to prevent reinfection, thereby further exacerbating bacterial resistance. Thus, it is imperative to develop novel biomaterials with dual antibacterial and bone regenerative functions to effectively treat jaw OM.

In recent years, artificial biomaterials have emerged as promising novel therapeutic agents for jaw OM owing to their excellent biocompatibility and bioactivity [12]. Among these advanced materials, metal–organic frameworks (MOFs) have stood out as a prominent class of porous crystalline materials composed of metal clusters and organic linkers [13], which offer enormous potential for pharmaceutical and biomedical applications, including antibacterial therapy [14], drug delivery [15], and anticancer treatment [16]. Notably, zeolitic imidazolate framework-8 (ZIF-8) represents a typical subclass of MOFs [17]. Its fundamental repeating unit is [ZnN_4_], where nitrogen atoms originate from imidazole groups, locally forming a structure in which each zinc atom is coordinated with four imidazole rings. These basic repeating units are bridged by 2-methylimidazole ligands, creating truncated octahedra. Subsequently, these octahedra further interconnect in three dimensions to construct a sodalite topology, a structure commonly observed in zeolites [18]. ZIF-8 integrates zeolite-like porosity, good biocompatibility, and a high specific surface area. It enables the rapid and sustained release of Zn^2+^ and encapsulated substances in low-pH environments, with extensive applications in functional materials and biomedical engineering [19]. Regarding the antibacterial activity of ZIF-8, its efficacy is primarily attributed to Zn^2+^ released upon its degradation [20]. Zn^2+^ attracts negatively charged bacteria via Coulombic interaction, subsequently binds to proteins and anions on the bacterial surface to disrupt cell membrane integrity, and ultimately leads to cytoplasmic leakage and bacterial death [21]. It can also interfere with the function of cytoplasmic proteins and nucleic acids, thereby disrupting bacterial metabolism [22]. Recently, studies have demonstrated that ZIF-8 inhibits the arginine biosynthesis pathway in bacteria, causing dysfunction of the tricarboxylic acid cycle and disruption of bacterial membrane integrity, ultimately eliminating MRSA [23]. Furthermore, in promoting osteogenesis, ZIF-8 can sequester calcium and phosphate ions from bodily fluids and undergo self-mineralization to form apatite-like crystals, enabling more precise mimicking of the natural bone tissue formation process [24]. Moreover, Zn^2+^ released from ZIF-8 degradation is considered an essential physiological trace mineral for the human body, as it plays a crucial role in bone physiology [25].

To enhance the biocompatibility, osteogenic properties, and targeted aggregability of ZIF-8, Fe_3_O_4_ NPs were incorporated into its structure. Fe_3_O_4_ NPs exhibit excellent biocompatibility, unique nanoscale biological effects, and can be taken up, expelled, and metabolized by cells [26]. Fe_3_O_4_ NPs also provide the dynamic mechanical stimulation needed for bone formation, directly promoting osteogenic differentiation of bone marrow mesenchymal stem cells (BMSCs) and osteogenesis in vivo, with broad applications in biomedical bone regeneration [27]. Crucially, the superparamagnetic properties of Fe_3_O_4_ NPs enable a response to a static magnetic field (SMF), thereby enhancing therapeutic efficacy via dual mechanisms: targeted aggregation and osteogenic synergy. Specifically, SMF itself accelerates the proliferation, migration, and differentiation of osteoblast-like cells, thereby promoting osteogenesis in BMSCs [28]. In synergy with SMF, Fe_3_O_4_ NPs can further alter cellular physicochemical properties and endocytosis, thus significantly boosting osteogenic-related behaviors and bone regeneration capacity [29].

Currently, Fe_3_O_4_@ZIF-8 NPs are mostly applied in reaction catalysis [30], biosensing [31], environmental adsorption [32], and drug delivery [33]. However, they are rarely used in antibacterial therapy, notably in the treatment of jaw OM. Polycaprolactone (PCL)/Fe_3_O_4_@ZIF-8 nanocomposite scaffolds have been shown to eliminate infections and promote new bone formation in infected bone defects [34]. However, they failed to explore the specific mechanisms underlying the scaffolds’ antibacterial activity, nor did they investigate the combined effect of the scaffolds with SMF. More recently, a core–shell structured therapeutic platform has been constructed with Fe_3_O_4_ as the core and ZIF-8 loaded with Au NPs and maltodextrin as the shell [35]. This core−shell structure enables the integration of various biofunctional materials, leveraging synergistic antibacterial pathways, including targeted binding, near-infrared (NIR)-induced hyperthermia, reactive oxygen species (ROS) generation, and Zn^2+^ release, to achieve efficient antibiosis and promote wound healing. Nevertheless, this platform has not been applied to infected bone defects, and its antibacterial performance is highly dependent on NIR irradiation. It has also failed to elucidate the specific antibacterial mechanism of Zn^2+^.

To address the dual clinical challenges of infection control and bone repair in jaw OM and overcome the limitations of existing Fe_3_O_4_@ZIF-8-based materials, we innovatively applied Fe_3_O_4_@ZIF-8 core–shell NPs to jaw OM therapy, leveraging the superparamagnetism of Fe_3_O_4_ and the pH-responsive degradation of ZIF-8. Subsequently, in vitro experiments were performed to evaluate the biocompatibility, osteogenic potential, and antibacterial activity of Fe_3_O_4_@ZIF-8 NPs, and to elucidate the nanoparticle-mediated antibacterial mechanism. Finally, a *S. aureus*-infected rat model of jaw OM was established to verify the in vivo therapeutic efficacy and biosafety of the nanoparticles.

## 2. Materials and Methods

### 2.1. Regents and Materials

All reagents were of analytical grade and used without additional purification. C_4_H_6_N_2_ (>98%), Zn(NO_3_)_2_∙6H_2_O (≥99%), methanol (99.5%), anhydrous ethanol (≥99.7%), FeCl_3_∙6H_2_O (≥99%), ethylene glycol (CH_2_OH)_2_ (≥99%), polyethylene glycol (PEG, molecular weight 20,000), and NaAc∙3H_2_O were commercially obtained from Anhui Zesheng Technology Co., Ltd. (Shanghai, China). Gelatin methacryloyl (GelMA) hydrogel was acquired from Engineering For Life (Suzhou, China). *o*-Nitrophenyl *β*-D-galactopyranoside (ONPG) was purchased from Beyotime Biotechnology Co., Ltd. (Shanghai, China). N,N,N’,N’-Tetrakis (2-pyridinylmethyl)-1,2-ethanediamine (TPEN) was obtained from Aladdin Biochemical Technology Co., Ltd. (Shanghai, China). Two NdFeB magnets with different specifications (30 mm × 20 mm × 5 mm, 150 mT; 30 mm × 20 mm × 10 mm, 280 mT) and one NdFeB magnetic patch (50 mm × 20 mm × 2 mm, 50 mT) were purchased from Ganzhou Dingxi Magnetic Industry Co., Ltd. (Ganzhou, China) and Shenzhen Cijiang Technology Co., Ltd. (Shenzhen, China), respectively. The intensity of the SMF generated by these magnets was measured and verified using a gaussmeter (Model KT-101, AIRUIPU, Shenzhen, China), thereby enabling the establishment of SMFs with different intensities as required for the study. *Staphylococcus aureus* (*S. aureus*, full species name: *Staphylococcus aureus* subsp. *aureus*, ATCC 25923) and *Escherichia coli* (*E. coli*, full species name: *Escherichia coli*, ATCC 25922) were obtained from the American Type Culture Collection (ATCC). *Streptococcus pyogenes* (*S. pyogenes*, full species name: *Streptococcus pyogenes* subsp. *pyogenes*, ATCC 19615) was acquired from Biofeng (Shanghai, China).

### 2.2. Synthesis of Fe_3_O_4_@ZIF-8 NPs

ZIF-8 NPs were successfully synthesized through the room-temperature solution method [36]. Briefly, 1314 mg of 2-methylimidazole (C_4_H_6_N_2_) and 594 mg of Zn(NO_3_)_2_·6H_2_O were each dispersed separately in 30 mL of methanol, followed by ultrasonication for 10 min to form clear solutions. Then, the two solutions were mixed by stirring, and the final mixture was stirred constantly at 25 °C for 4 h. Following centrifugation at 10,000 rpm for 10 min, the supernatant was removed, and the resulting precipitate was rinsed three times with anhydrous ethanol and deionized water to remove unreacted starting materials and impurities. Subsequently, the product was dried overnight in a vacuum oven at 60 °C. The resulting white powder was identified as ZIF-8 NPs.

According to previously published literature, Fe_3_O_4_ NPs were fabricated by a solvothermal approach [37]. In brief, 1.35 g FeCl_3_·6H_2_O, 1.0 g PEG, and 3.6 g NaAc·3H_2_O were solubilized in 40 mL ethylene glycol with intense stirring, yielding a uniform solution. The resulting mixture was transferred to a Teflon-lined autoclave, which was then maintained at 200 °C for 72 h to allow the reaction to proceed. Upon cooling to 25 °C, the black precipitate was harvested by magnetic separation, washed three times with anhydrous ethanol and deionized water, and subsequently dried in a vacuum oven at 60 °C for 12 h. The obtained black powder was verified to be Fe_3_O_4_ NPs.

Fe_3_O_4_@ZIF-8 NPs were synthesized via a coprecipitation method [38]. Briefly, 1.8 g Zn(NO_3_)_2_·6H_2_O was solubilized in 30 mL methanol; subsequently, 0.1 g of pre-synthesized Fe_3_O_4_ NPs was introduced into the solution, and the mixture was ultrasonically treated for 15 min. Next, 30 mL of a methanol solution containing 3.9 g 2-methylimidazole (C_4_H_6_N_2_) was added, and the mixture was further ultrasonicated for 15 min to achieve a homogeneous system. The reaction mixture was then transferred to a three-neck flask and vigorously stirred at 25 °C for 4 h. The composite precipitate was separated by external magnetic separation, alternately washed three times with anhydrous ethanol and deionized water to remove unreacted precursors and impurities, and finally dried overnight in a vacuum oven at 60 °C. The obtained brown powder was identified as Fe_3_O_4_@ZIF-8 NPs.

### 2.3. Instruments for Fe_3_O_4_@ZIF-8 NPs Characterization

The morphologies of ZIF-8, Fe_3_O_4_, and Fe_3_O_4_@ZIF-8 NPs were observed by transmission electron microscopy (TEM, FEI Talos F200x, Hillsboro, OR, USA) at 120 kV. The hydrodynamic diameters and zeta potentials of ZIF-8, Fe_3_O_4_, and Fe_3_O_4_@ZIF-8 NPs in PBS were measured by dynamic light scattering (DLS, Malvern Zetasizer Pro, Worcestershire, UK) at 25 °C. The crystal structure of the nanoparticles was determined by X-ray diffraction (XRD, Rigaku SmartLab SE, Yokyo, Japan) with a Cu target operating at 40 kV and 100 mA over a 2θ range of 10–80°. The elemental composition and chemical states of ZIF-8, Fe_3_O_4_, and Fe_3_O_4_@ZIF-8 NPs were analyzed using X-ray photoelectron spectroscopy (XPS, Thermo Scientific K-Alpha, Waltham, MA, USA). Their surface morphologies and elemental distributions were examined by scanning electron microscopy (SEM, ZEISS Sigma 300, Baden-Württemberg, Germany) and energy-dispersive X-ray spectrometry (EDS, OXFORD Xplore, England, UK), respectively. The thermal stabilities of ZIF-8 and Fe_3_O_4_@ZIF-8 NPs were evaluated via thermogravimetric analysis (TGA, Mettler Toledo TGA/DSC3+, Zurich, Switzerland) under a nitrogen atmosphere. TGA was conducted at a heating rate of 10 °C/min over the temperature range of 30 °C to 1500 °C. The magnetic properties of Fe_3_O_4_ and Fe_3_O_4_@ZIF-8 NPs were determined using a vibrating sample magnetometer (VSM, LakeShore 7410, Westerville, OH, USA). The concentrations of Zn^2+^ and Fe^2+^/Fe^3+^ released from Fe_3_O_4_@ZIF-8 NPs were quantified using inductively coupled plasma optical emission spectroscopy (ICP-OES, Agilent 5110, Santa Clara, CA, USA).

### 2.4. Isolation and Culture of BMSCs

All studies involving Sprague–Dawley (SD) rats complied with animal ethics standards approved by the Research Ethics Committee of The Fourth Military Medical University (Approval No. 20250162, Shaanxi, China). Healthy 2-week-old male SD rats (40–60 g) were euthanized via cervical dislocation to collect bone marrow from femurs and tibias, and BMSCs were cultured via the whole bone marrow adhesion method, as described and validated in a prior study [39]. Subsequently, the cells were cultured in Dulbecco’s Modified Eagle Medium (DMEM, Gibco, Gaithersburg, MD, USA) supplemented with 10% fetal bovine serum (FBS, Gibco) and 1% penicillin-streptomycin solution (Gibco) in a 5% CO_2_ incubator maintained at 37 °C. The culture medium was replenished every 3 days. Cells from passages 3 to 5 were used for subsequent experiments to ensure consistent viability and phenotypic stability. Flow cytometry was used to detect surface markers (CD29, CD90, CD34, and CD45) for the identification of BMSCs.

### 2.5. CCK-8 Assays and Live/Dead Cell Staining

To evaluate the in vitro biocompatibility of Fe_3_O_4_@ZIF-8 NPs, the CCK-8 assay was conducted in two steps: First, the optimal Fe_3_O_4_@ZIF-8 NPs concentration was screened, and the optimal SMF intensity was determined based on this concentration. BMSCs were seeded in 96-well plates at a density of 5 × 10^3^ cells/well and incubated for 12 h to allow for adhesion. To determine the optimal Fe_3_O_4_@ZIF-8 NPs concentration, cells were treated with gradient concentrations of Fe_3_O_4_@ZIF-8 NPs (0, 25, 50, 100, 125, 150, 175, and 200 µg/mL). After 24, 48, and 72 h of co-culture, supernatants were aspirated, fresh medium with 10% CCK-8 reagent (Beyotime, Shanghai, China) was added, and after an incubation period of 2 h at 37 °C, the optical density at 450 nm (OD450) was determined using a microplate reader (Epoch, Winooski, VT, USA) to confirm the optimal nanoparticle concentration. Subsequently, under the same seeding conditions, the cells were co-cultured with the predetermined optimal nanoparticle concentration and treated with gradient SMF intensities (0, 50, 100, and 200 mT). The cell viability was assessed using the same CCK-8 protocol at the aforementioned time points to determine the optimal SMF intensity.

To further validate these optimal conditions, BMSCs were fluorescently stained using a Calcein/PI Cell Viability/Cytotoxicity Assay Kit (Beyotime, Shanghai, China). Specifically, BMSCs were inoculated into laser confocal dishes at two densities: 1 × 10^5^ cells/dish for the optimal nanoparticle concentration experiment; 1 × 10^4^ cells/dish for the optimal SMF intensity experiment (based on the pre-identified nanoparticle concentration). After 72 h of co-culture, BMSCs were stained and detected using the aforementioned kit, strictly following the manufacturer’s instructions. The stained cells were visualized using a confocal laser-scanning microscope (CLSM; Olympus, Tokyo, Japan).

### 2.6. ALP Staining, ALP Activity Measurement, and ARS Staining

BMSCs were plated in six-well plates at a density of 2 × 10^5^ cells/mL and maintained in a 5% CO_2_ incubator at 37 °C. After a 12 h adhesion period, the culture medium was replaced with osteogenic induction medium supplemented with 100 nM dexamethasone, 50 mM vitamin C, and 10 mM glycerophosphate (Beyotime, Shanghai, China). At 7 and 14 days post-induction, alkaline phosphatase (ALP) staining was performed using a BCIP/NBT ALP Color Development Kit (Beyotime, Shanghai, China). ALP activity in the supernatant was determined using an ALP activity kit (Jiancheng Inc., Nanjing, China) in accordance with the manufacturer’s protocol, and the absorbance at 520 nm was measured using a microplate reader (Epoch, Winooski, VT, USA). Alizarin red S (ARS) staining was conducted using a 0.1% Alizarin Red S solution (Solarbio, Beijing, China) after 21 days of osteogenic induction. To quantify extracellular matrix calcification, the stained cells were destained with 10% cetylpyridinium chloride dissolved in 10 mM sodium phosphate, and the absorbance at 562 nm was measured using a microplate reader (Epoch, Winooski, VT, USA).

### 2.7. RT-qPCR

Total cellular RNA was extracted using a total RNA extraction kit (Omega Bio-tek, Norcross, GA, USA) according to the manufacturer’s protocol, and reverse transcribed into cDNA using the PrimeScript^TM^ RT Master Mix kit (Takara, Shiga, Japan) following the manufacturer’s instructions. The expression levels of *Alp*, osteocalcin (*Ocn*), and runt-related transcription factor 2 (*Runx2*) were quantified using an Applied Biosystems 7500 Real-Time PCR System (Thermo Fisher Scientific, Waltham, MA, USA) with a SYBR Green-based real-time PCR assay. Glyceraldehyde-3-phosphate dehydrogenase (GAPDH) served as the housekeeping gene. Primer sequences are listed in Table S1. Relative fold-changes in gene expression were determined using the 2^−∆∆CT^ method.

### 2.8. Western Blot

Total cell proteins were isolated with RIPA buffer (Zhonghuihecai, Xi’an, Shaanxi, China) supplemented with a 1% protease inhibitor. Protein samples were boiled in loading buffer (Omega Bio-tek, USA), separated by 10% SDS-PAGE at a constant 160 V for 40 min, and transferred to polyvinylidene fluoride (PVDF) membranes (Millipore, Burlington, MA, USA) under a constant current of 400 mA for 30 min. PVDF membranes were blocked using a 5% non-fat dry milk solution, followed by overnight incubation at 4 °C with primary antibodies targeting GAPDH (1:5000, HRP-60004, Proteintech, Chicago, IL, USA), ALP (1:500, ab65834, Abcam, Cambridge, UK), OCN (1:1000, DF12303, Affinity, Cincinnati, OH, USA), and RUNX2 (1:1000, GTX00792, GeneTex, Irvine, CA, USA). After three rounds of washing with TBST (Proandy, Xi’an, Shaanxi, China), the membranes were incubated with HRP-linked secondary antibodies (1:5000, GeneTex, Irvine, CA, USA) at room temperature for 1 h. The protein bands were detected using a chemiluminescence imaging system (Bio-Rad, Hercules, CA, USA). The relative expression levels of target proteins were normalized to those of GAPDH.

### 2.9. Bacterial Culture

*S. aureus*, *S. pyogenes*, and *E. coli* were used to evaluate the antibacterial activity of the Fe_3_O_4_@ZIF-8 NPs. Prior to the experiment, *S. aureus* and *E. coli* were incubated in Luria–Bertani (LB) medium at 37 °C for 12 h, whereas *S. pyogenes* was cultured in brain heart infusion (BHI) medium at the same temperature. After culturing, the bacterial cultures were centrifuged, and the pellets were washed three times with phosphate-buffered saline (PBS), then diluted to a final concentration of 10^7^ CFU/mL for subsequent use. For antibacterial evaluation, *S. aureus* and *E. coli* were inoculated onto LB agar plates, whereas *S. pyogenes* was inoculated onto BHI agar plates. All experimental groups were subjected to 100 mT SMF stimulation for all antibacterial activity assays.

### 2.10. Determination of Minimum Inhibitory Concentration

First, 100 µL of Fe_3_O_4_@ZIF-8 NPs at different concentrations (0, 25, 50, 100, 200, and 400 µg/mL) was added to the wells of a sterile 96-well plate. An antibiotic control group served as the positive control. Each group consisted of three parallel replicates. Next, 10 µL of the bacterial suspension (10^7^ CFU/mL) was added to each well. The 96-well plate was subsequently transferred to an incubator and maintained at 37 °C for 16 h. After the incubation period, 10 µL of 0.0625% resazurin solution was added to each well, and the mixture was incubated at 37 °C for an additional 2 h. Finally, the antibacterial effect was evaluated based on the color change of the solution in each well.

### 2.11. The CFU Counting Assay

The antibacterial effects against each bacterial strain were assessed using a colony-forming unit (CFU) counting assay. Briefly, 10 µL of a diluted bacterial suspension (10^7^ CFU/mL) was resuspended in 100 µL of a medium containing Fe_3_O_4_@ZIF-8 NPs at final concentrations of 0, 25, 50, 100, and 200 µg/mL. After incubation at 37 °C for 16 h, the mixed solution from each group was diluted to the appropriate concentration. After spreading 100 µL of each diluted bacterial suspension onto the agar plates, the plates were incubated for an additional 24 h before the CFU were counted. Each test was repeated three times.

### 2.12. Bacterial Live/Dead Staining

The antibacterial effects of Fe_3_O_4_@ZIF-8 NPs against *S. aureus*, *S. pyogenes*, and *E. coli* were evaluated using a LIVE/DEAD Bacterial Staining Kit (APExBIO Technology LLC, Houston, TX, USA). Briefly, 1 mL of bacterial suspension (10^6^ CFU/mL) was incubated for 24 h, followed by treatment with Fe_3_O_4_@ZIF-8 NPs at final concentrations of 0, 25, 50, 100, and 200 µg/mL. After incubation for 2 h, NucGreen and EthD-III fluorescent dyes were added to the samples, followed by an additional 30 min incubation in the dark prior to observation using CLSM (Olympus, Tokyo, Japan).

### 2.13. Crystal Violet Staining of Bacterial Biofilm

To evaluate the inhibitory effect of the Fe_3_O_4_@ZIF-8 NPs on biofilm formation, crystal violet staining was performed. Briefly, 1 mL of 10^6^ CFU/mL bacterial suspension was cultured in a sterile 24-well plate for 24 h to form biofilms. Then, 1 mL of medium containing Fe_3_O_4_@ZIF-8 NPs at various concentrations was transferred to each well, followed by incubation for 16 h. The biofilms were washed three times with PBS to remove residual culture medium, fixed with glutaraldehyde for 30 min, stained with 0.1% crystal violet for 30 min, and washed to remove unbound dye prior to drying. Subsequently, the stained biofilms were dissolved in 200 µL of 75% ethanol solution, and the optical absorbance at 570 nm was detected using a microplate reader (Epoch, Winooski, VT, USA).

### 2.14. Bacterial Membrane Permeability

The permeability of bacterial membranes was evaluated using an ONPG hydrolysis experiment. First, 100 µL of culture medium containing different concentrations of Fe_3_O_4_@ZIF-8 NPs was added to the wells of a sterile 96-well plate, followed by 10 µL of the bacterial suspension (10^7^ CFU/mL). The mixture was then incubated for 16 h. Subsequently, 25 µL of ONPG solution (4 mg/mL) was added to each well, and incubation was continued for another 30 min. Next, 25 µL of 1 mol/L sodium carbonate (Na_2_CO_3_) was added to terminate the reaction, and the optical absorbance of the solution at 420 nm was promptly determined using a microplate reader (Epoch, Winooski, VT, USA).

### 2.15. Protein Leakage

A protein concentration standard curve was constructed using a bicinchoninic acid (BCA) protein assay kit (Zhonghuihecai, Xi’an, Shaanxi, China). Briefly, 10 µL of bacterial suspension (10^7^ CFU/mL) was mixed with 100 µL of medium containing different concentrations of Fe_3_O_4_@ZIF-8 NPs and incubated at 37 °C for 16 h. The bacterial suspension was then collected and centrifuged at 8000 rpm for 5 min. Subsequently, 20 µL of the supernatant was pipetted into a 96-well plate, followed by the addition of 200 µL of BCA working solution. After 30 min of incubation at 37 °C, the optical absorbance of the mixture at 562 nm was determined with a microplate reader (Epoch, Winooski, VT, USA).

### 2.16. The Morphological Changes of Bacteria

The morphological changes of *S. aureus*, *S. pyogenes*, and *E. coli* in the different samples were examined using SEM. In brief, 1 mL of diluted bacterial suspension (10^6^ CFU/mL) was incubated at 37 °C for 24 h. After aspirating the medium, 1 mL of medium containing either 0 µg/mL (control group) or 100 µg/mL Fe_3_O_4_@ZIF-8 NPs was added to the respective wells. After 2 h of co-incubation, the samples were fixed overnight in 2.5% glutaraldehyde at 4 °C, rinsed with deionized water, and sequentially dehydrated using a graded ethanol series (50%, 75%, 90%, and 95% for 15 min each; 100% for 30 min). The specimens were then vacuum-dried and sputter-coated with gold before SEM imaging (ZEISS Sigma 300, Baden-Württemberg, Germany).

### 2.17. Intrabacterial Zn^2+^ Detection

After 16 h of co-cultivation of bacteria under the different experimental treatments, the mixture was centrifuged. The resulting bacterial pellet was digested with 65% (*v*/*v*) nitric acid (HNO_3_) at 90–95 °C for 4 h, and the concentration of Zn^2+^ in the digested solution was quantified using ICP-OES (Agilent 5110, Santa Clara, CA, USA).

### 2.18. Transcriptome Analysis

*S. aureus* (10^8^ CFU/mL) was first inoculated into LB medium and incubated at 37 °C for 6 h. Subsequently, the bacteria in the NPs group were transferred to LB medium containing 100 µg/mL Fe_3_O_4_@ZIF-8 NPs and exposed to 100 mT SMF stimulation. In contrast, those in the control group were solely transferred to fresh LB medium under the same SMF stimulation condition. Both groups were then further incubated at 37 °C for 16 h. Following this incubation, transcriptome analysis was performed on *S. aureus* from both groups. Total RNA extraction, RNA quality detection, and RNA sequencing were performed by Majorbio Co., Ltd. (Shanghai, China). Primers for subsequent RT-qPCR assays were designed and synthesized by Sangon Biotech (Shanghai, China), and their sequences are shown in Table S2. The PrimeScript FAST RT Reagent Kit with gDNA Eraser (Takara, Japan) and PrimeScript RT Master Mix (Takara, Japan) were used for RT-qPCR. The relative mRNA expression levels were normalized to those of 16S rRNA. Western blot was performed to assess the protein expression levels of DnaK (1:5000, ab69617, Abcam) and GroEL (1:5000, ab318970, Abcam). The relative expression levels of target proteins were normalized to those of RpoB (1:5000, ab191598, Abcam).

### 2.19. Establishment of an S. aureus-Infected Jaw Osteomyelitis Rat Model

Healthy 8-week-old male SD rats (250–300 g) were anesthetized with an intraperitoneal injection of 3% pentobarbital sodium (Sigma-Aldrich, St. Louis, MO, USA) at a dose of 80 mg/kg body weight. Surgical preparation (skin preparation, disinfection, and draping) was performed. A 15 mm full-thickness longitudinal incision was made parallel to the inferior border of the left mandible. Subcutaneous tissue, deep fascia, and periosteum were dissected to expose the mandible. A 4 mm diameter full-thickness defect was created on the left mandible using a trephine bur. The defect was made on the buccal side, inferior to the incisor root and posterior to the second molar, sparing the mandibular borders. The model group received 20 µL of 10^7^ CFU/mL *S. aureus* into the defect, and the control group received 20 µL of PBS at the same site (four rats in each group). Both were covered with fibrin glue to prevent leakage, followed by layered muscle and skin sutures. Two weeks postoperatively, rats were sacrificed via intraperitoneal injection of 3% pentobarbital sodium at 250 mg/kg body weight for validation of the *S. aureus*-infected jaw OM rat model, and subsequent detection of various indicators was performed using the collected samples. Soft tissues were collected from the defect areas for bacterial load quantification. The left mandibular bones were excised, and micro-computed tomography (micro-CT) was used to acquire bone tissue data to analyze and calculate the bone volume/tissue volume fraction (BV/TV), trabecular thickness (Tb.Th), bone mineral density (BMD), bone surface to tissue volume (BS/TV), and trabecular separation (Tb.Sp). After decalcification, Hematoxylin-eosin (H&E), Masson, and Giemsa staining were performed to evaluate bone regeneration, inflammation, and infection in the defect area.

### 2.20. In Vivo Therapy of Fe_3_O_4_@ZIF-8 NPs on S. aureus-Infected Jaw Osteomyelitis

After establishing the *S. aureus*-infected jaw OM rat model, all rats underwent debridement of the infected area: pus, infected granulation tissue, and free sequestra were fully removed without damaging the mandible. The rats were randomly divided into three groups (four rats per group at each time point).

(i) GM group: 20 µL of 10% (*w*/*v*) GelMA hydrogel was injected into the bone defect after debridement;
(ii) GM/NPs group: 20 µL of 10% (*w*/*v*) GelMA hydrogel loaded with 100 µg/mL Fe_3_O_4_@ZIF-8 NPs was injected post-debridement;
(iii) GM/NPs + SMF group: the same hydrogel as in the GM/NPs group with an additional 100 mT SMF stimulation postoperatively.

The hydrogels were UV-cured, and the incisions were sutured tightly. At 4 and 8 weeks post-treatment, the rats were euthanized as previously described, and samples were collected and evaluated by gross observation, micro-CT, and histological staining.

### 2.21. Statistical Analysis

All experiments were repeated at least three times per group. The results were analyzed using GraphPad Prism software (version 9.5.0, USA) and were presented as mean ± standard deviation (SD). Statistical differences were determined using an unpaired *t*-test (for two groups) and one-way analysis of variance (ANOVA) with Tukey’s post hoc test (for three or more groups). The following values were considered to be statistically significant: * *p* < 0.05, ** *p* < 0.01, *** *p* < 0.001, and **** *p* < 0.0001, and ns: no significance.

## 3. Results and Discussion

### 3.1. Characterization of Fe_3_O_4_@ZIF-8 NPs

**Figure 1:**
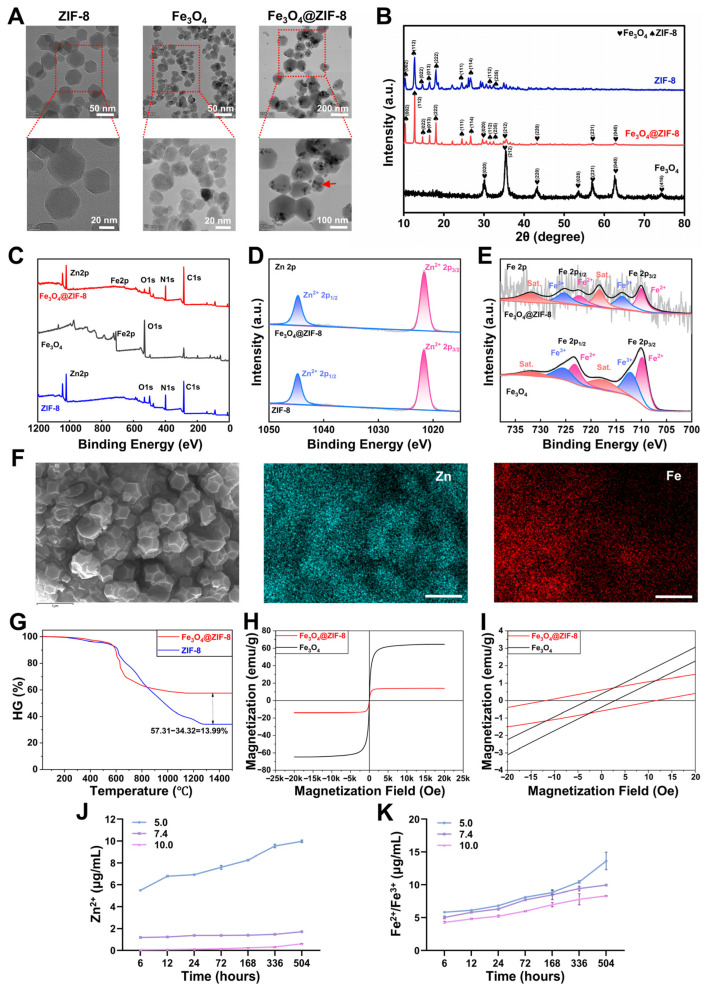
Characterization of Fe_3_O_4_@ZIF-8 NPs. (**A**): TEM images of ZIF-8, Fe_3_O_4_, and Fe_3_O_4_@ZIF-8 NPs (red arrow: Fe_3_O_4_ NPs); (**B**,**C**): XRD patterns and XPS of ZIF-8, Fe_3_O_4_, and Fe_3_O_4_@ZIF-8 NPs; (**D**): XPS spectra of Zn 2p in ZIF-8 and Fe_3_O_4_@ZIF-8 NPs; (**E**): XPS spectra of Fe 2p in Fe_3_O_4_ and Fe_3_O_4_@ZIF-8 NPs; (**F**): EDS mapping images of Fe_3_O_4_@ZIF-8 NPs; (**G**): TGA results of ZIF-8 and Fe_3_O_4_@ZIF-8 NPs. (**H**,**I**): Magnetization curves and magnetic behavior of Fe_3_O_4_ and Fe_3_O_4_@ZIF-8 NPs under a magnetic field; (**J**,**K**): The release curve of Zn^2+^ and Fe^2+^/Fe^3+^ from Fe_3_O_4_@ZIF-8 NPs under different pH conditions over time.

In our study, Fe_3_O_4_@ZIF-8 core–shell nanoparticles (NPs) were successfully synthesized via a coprecipitation method (Figures S1 and S2). Transmission electron microscopy (TEM) analysis revealed that the zeolitic imidazolate framework-8 (ZIF-8) crystals exhibited a hexagonal shape with a diameter of 60.27 ± 4.82 nm, and Fe_3_O_4_ NPs were spherical with a diameter of 24.06 ± 3.58 nm. Fe_3_O_4_@ZIF-8 NPs exhibited a hexagonal shape consistent with that of ZIF-8 NPs (serving as the shell), with spherical Fe_3_O_4_ NPs (acting as the core, emphasized by a red arrow) tightly embedded within ZIF-8, forming a distinct core–shell structure and a diameter of 158.76 ± 9.31 nm (Figure 1A and Figure S3). Dynamic light scattering (DLS) analysis revealed that the hydrodynamic diameters of ZIF-8, Fe_3_O_4_, and Fe_3_O_4_@ZIF-8 NPs were 81.2 nm, 34.82 nm, and 168.3 nm, respectively (Figure S4A). Meanwhile, the zeta potential measurements indicated that these three types of nanoparticles exhibited negative surface charges, with values of −21.89 mV, −30.48 mV, and −11.16 mV for ZIF-8, Fe_3_O_4_, and Fe_3_O_4_@ZIF-8 NPs, respectively (Figure S4B). The negative zeta potential of Fe_3_O_4_@ZIF-8 NPs ensured effective electrostatic repulsion, preventing particle agglomeration and conferring favorable colloidal stability in aqueous systems. The crystal structures of ZIF-8, Fe_3_O_4_, and Fe_3_O_4_@ZIF-8 NPs were determined using X-ray diffraction (XRD). The characteristic peaks of Fe_3_O_4_ NPs appeared in the XRD pattern of Fe_3_O_4_@ZIF-8 NPs, which matched magnetite (JCPDS No. 19-0629) [40], indicating successful incorporation of Fe_3_O_4_ NPs. Fe_3_O_4_@ZIF-8 NPs exhibited an XRD pattern similar to that of ZIF-8 NPs [41], confirming that the sodalite structure of ZIF-8 NPs remained intact after Fe_3_O_4_ NPs loading. These results confirmed the successful formation of Fe_3_O_4_@ZIF-8 NPs from both components (Figure 1B). X-ray photoelectron spectroscopy (XPS) was used to characterize the compositions of the ZIF-8, Fe_3_O_4_, and Fe_3_O_4_@ZIF-8 NPs. Fe_3_O_4_@ZIF-8 NPs displayed the characteristic elemental peaks of C, N, O, Fe, and Zn (Figure 1C). Fe_3_O_4_@ZIF-8 NPs not only retained the Fe 2p and O 1s peaks corresponding to Fe_3_O_4_ NPs but also incorporated the Zn 2p, N 1s, and C 1s peaks characteristic of ZIF-8 NPs. Specifically, the binding energies of the Zn 2p peaks at 1021.7 and 1044.7 eV could be assigned to Zn^2+^ 2p_3/2_ and Zn^2+^ 2p_1/2_, which matched the characteristic signals of Zn in ZIF-8, confirming the successful formation of ZIF-8 NPs in Fe_3_O_4_@ZIF-8 NPs (Figure 1D) [42]. In the Fe 2p spectrum (Figure 1E), two distinct peaks at 713.9 and 725.3 eV were assigned to Fe^3+^ 2p_3/2_ and Fe^3+^ 2p_1/2_, whereas those at 710.0 and 722.4 eV corresponded to Fe^2+^ 2p_3/2_ and Fe^2+^ 2p_1/2_, respectively, consistent with the Fe valence state in Fe_3_O_4_ NPs, confirming that Fe in Fe_3_O_4_@ZIF-8 NPs exists as Fe_3_O_4_ [43]. These coordinated characteristic peaks and element signals directly confirmed the successful composite formation of Fe_3_O_4_ and ZIF-8 NPs.

Energy-dispersive X-ray spectrometry (EDS) was employed to determine the elemental distribution of Fe_3_O_4_@ZIF-8 NPs. The results demonstrated that C, N, O, Fe, and Zn elements were uniformly distributed in the Fe_3_O_4_@ZIF-8 NPs (Figure 1F and Figure S5), with the mass fraction of Fe element measured as 22.59% (Table S3). Based on the mass ratio of Fe in Fe_3_O_4_ (72.36%), the loading content of Fe_3_O_4_ was calculated to be approximately 31.22%. Thermogravimetric analysis (TGA) results showed that both Fe_3_O_4_@ZIF-8 and ZIF-8 NPs were stable below 600 °C. Fe_3_O_4_@ZIF-8 NPs exhibited a sharp weight loss at approximately 647 °C, which was slightly higher than the corresponding weight loss temperature of ZIF-8 NPs (635 °C) (Figure 1G). The mass loss in ZIF-8 NPs primarily resulted from the decomposition of organic ligands. This increase in the thermal decomposition temperature and reduction in the mass loss rate suggested that the incorporation of Fe_3_O_4_ NPs enhanced the thermal stability of the ZIF-8 framework. Moreover, based on the mass loss calculations for Fe_3_O_4_@ZIF-8 and ZIF-8 NPs, the loading content of Fe_3_O_4_ NPs in the ZIF-8 crystals was estimated to be 13.99%. The quantitative difference between EDS and TGA results arose from their distinct characterization principles: EDS is a semi-quantitative surface elemental analysis, while TGA relies on organic ligand thermal decomposition [30]. Their consistent confirmation of Fe_3_O_4_ loading further validated the successful fabrication of the Fe_3_O_4_@ZIF-8 core–shell structure.

Magnetization curves showed that the saturation magnetization of Fe_3_O_4_@ZIF-8 and Fe_3_O_4_ NPs was 13.98 emu/g and 64.55 emu/g, respectively (Figure 1H). The incorporation of Fe_3_O_4_ NPs endowed Fe_3_O_4_@ZIF-8 NPs with excellent magnetic properties by enhancing their saturation magnetization. Additionally, the low coercivity and remanence indicated that Fe_3_O_4_@ZIF-8 NPs exhibited superparamagnetism, facilitating their rapid magnetic response (Figure 1I and Figure S6). To evaluate Zn^2+^ and Fe^2+^/Fe^3+^ release from Fe_3_O_4_@ZIF-8 NPs, cumulative release experiments were conducted in simulated body fluid at 37 °C and pH 5.0, 7.4, and 10.0, simulating acidic, neutral, and alkaline conditions, respectively. For Zn^2+^ release (Figure 1J), 5.48, 1.18, and 0.05 µg/mL were released at 6 h under pH 5.0, 7.4, and 10.0, respectively. Sustained slow release occurred over 21 days at pH 7.4 and 10.0, whereas a notable burst release was observed at pH 5.0 over 21 days. This pH-dependent burst release may be attributed to enhanced degradation of the ZIF-8 framework in acidic environments, thereby enabling continuous Zn^2+^ release. For Fe^2+^/Fe^3+^ release (Figure 1K), the concentrations were approximately 5 µg/mL across all pH conditions at 6 h, indicating an initial consistent release behavior. Over the subsequent 21 days, all pH groups exhibited a sustained slow release, except for a burst release at pH 5.0 during 14–21 days. These results collectively confirmed the pH-dependent release of Zn^2+^ and Fe^2+^/Fe^3+^ from Fe_3_O_4_@ZIF-8 NPs.

### 3.2. In Vitro Biocompatibility of Fe_3_O_4_@ZIF-8 NPs

**Figure 2:**
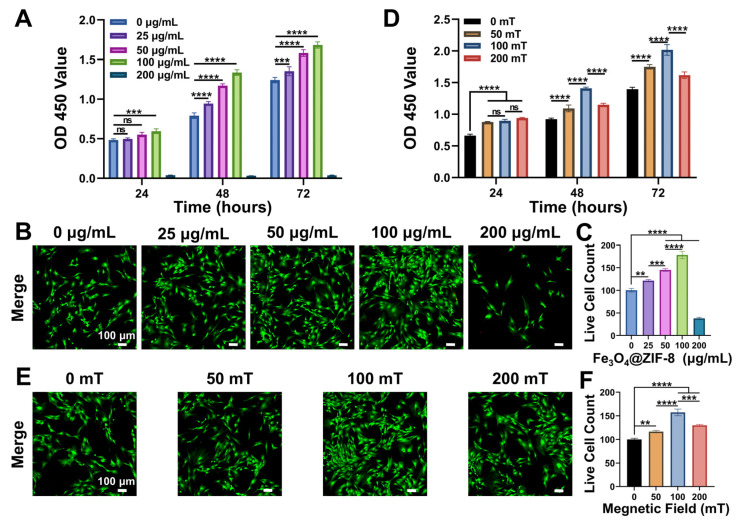
In vitro biocompatibility of Fe_3_O_4_@ZIF-8 NPs. (**A**): CCK-8 assay of BMSCs co-cultured with Fe_3_O_4_@ZIF-8 NPs at various concentrations for 24, 48, and 72 h; (**B**,**C**): Live/dead cell staining fluorescence images and quantification of BMSCs co-cultured with Fe_3_O_4_@ZIF-8 NPs at various concentrations for 72 h; (**D**): CCK-8 assay of BMSCs co-cultured with 100 µg/mL Fe_3_O_4_@ZIF-8 NPs and different SMF intensities for 24, 48, and 72 h; (**E**,**F**): Live/dead cell staining fluorescence images and quantification of BMSCs co-cultured with 100 µg/mL Fe_3_O_4_@ZIF-8 NPs and different SMF intensities for 72 h. *n* = 3, ns: no significance, ** *p* < 0.01, *** *p* < 0.001, and **** *p* < 0.0001.

Biocompatibility and osteogenic properties are critical for evaluating bone repair biomaterials, and an optimal microenvironment for cell survival is vital for in vitro assessment of magnetic materials [44]. In this study, cultured bone marrow mesenchymal stem cells (BMSCs) were identified by flow cytometry, which showed positivity for surface markers CD29 (98.9%) and CD90 (98.6%) and negativity for CD34 (1.5%) and CD45 (1.8%) (Figure S7). To investigate the effect of different Fe_3_O_4_@ZIF-8 NPs concentrations on BMSCs, BMSCs were co-cultured with 0, 25, 50, 100, and 200 µg/mL Fe_3_O_4_@ZIF-8 NPs and analyzed using CCK-8 assays and live/dead cell staining. As shown in Figure 2A, the CCK-8 assay results revealed that at all time points, the proliferation rate of BMSCs in the 100 µg/mL group was significantly higher than that in the other groups, whereas that in the 200 µg/mL group was significantly lower than that in the other groups. To further clarify the concentration-dependent effect of Fe_3_O_4_@ZIF-8 NPs on BMSCs’ proliferation, additional intermediate concentrations (125, 150, and 175 μg/mL) were supplemented between 100 and 200 μg/mL. The results showed that the cell proliferation rate peaked at 100 μg/mL, decreased slightly at 125 μg/mL, and declined significantly at 150–200 μg/mL (Figure S8). The enhanced proliferation at 100 μg/mL was primarily ascribed to the synergistic effects of moderately released Zn^2+^ and Fe_3_O_4_ NPs: Zn^2+^, as an essential trace element, activated Cyclin D1 and MAPK/ERK pathway to promote cell cycle progression, [45] while Fe_3_O_4_ NPs-induced magnetic mechanical stimulation boosted Ca^2+^ influx and mitochondrial metabolism, supporting cell proliferation [46,47]. In contrast, the decreased cell viability at concentrations > 100 μg/mL was due to excessive Zn^2+^-induced ionic imbalance [48], aggregation-dependent lysosomal disruption [49], and metabolic burden from excessive endocytosis [50]. As shown in Figure 2B,C, few or no dead cells were observed in all groups at 72 h. Notably, the number of viable cells in the 100 µg/mL group was significantly higher than that in the other groups, whereas that in the 200 µg/mL group was significantly lower than that in the other groups. Thus, these results indicated that 100 µg/mL Fe_3_O_4_@ZIF-8 NPs exhibited excellent biocompatibility, and this concentration was selected as the optimal concentration for subsequent experiments.

A static magnetic field (SMF) is also a critical factor for BMSCs co-cultured with Fe_3_O_4_@ZIF-8 NPs and plays a vital role in bone tissue regeneration [29]. To explore the optimal SMF intensity for BMSCs treated with 100 µg/mL Fe_3_O_4_@ZIF-8 NPs, we used a designed magnetic device (Figure S9) with different intensities of 0, 50, 100, and 200 mT. CCK-8 results (Figure 2D) showed that the proliferation rate of BMSCs in the 100 mT group was significantly higher than that in the other SMF groups at all time points. The live/dead cell staining results (Figure 2E,F) further confirmed that the number of viable BMSCs in the 100 mT group was significantly higher than that in the other SMF groups. Taken together, the combination of 100 µg/mL Fe_3_O_4_@ZIF-8 NPs and 100 mT SMF stimulation not only ensured excellent biocompatibility but also synergistically promoted the proliferation and viability of BMSCs. Thus, this combination was selected as the optimal treatment for subsequent in vitro osteogenic induction experiments with BMSCs.

### 3.3. In Vitro Osteogenic Effect of Fe_3_O_4_@ZIF-8 NPs

**Figure 3:**
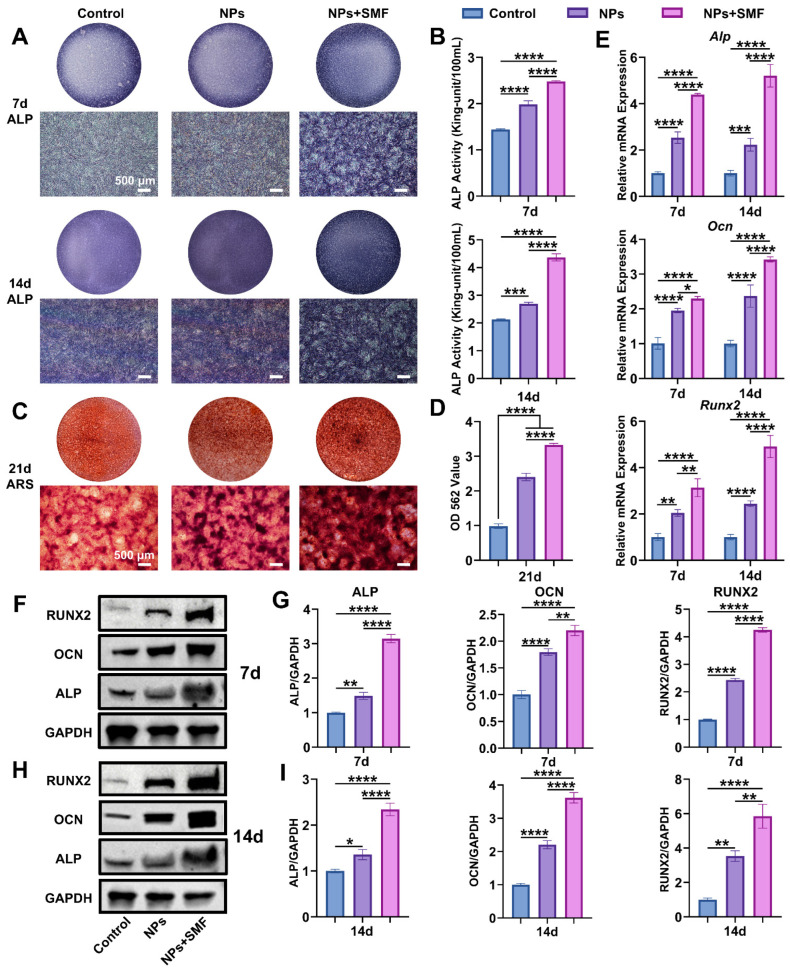
In vitro osteogenic effect of Fe_3_O_4_@ZIF-8 NPs. (**A**,**B**): ALP staining and quantification of ALP activity after osteogenic induction for 7 and 14 days; (**C**,**D**): ARS staining and semi-quantitative analysis after 21 days of osteogenic induction; (**E**): Relative mRNA expression levels of osteogenesis-related genes (*Alp*, *Ocn*, and *Runx2*) after induction for 7 and 14 days; (**F**–**I**): Western blot and quantification of osteogenesis-related proteins (ALP, OCN, and RUNX2) after induction for 7 (**F**,**G**) and 14 (**H**,**I**) days. *n* = 3, * *p* < 0.05, ** *p* < 0.01, *** *p* < 0.001, and **** *p* < 0.0001.

Good osteogenic differentiation ability is essential for bone regeneration materials [51]. To evaluate the osteogenic capacity of Fe_3_O_4_@ZIF-8 NPs combined with SMF stimulation in vitro, BMSCs were cultured in osteogenic induction medium and divided into three groups: the control group (no NPs and SMF), the NPs group (100 µg/mL Fe_3_O_4_@ZIF-8 NPs), and the NPs + SMF group (100 µg/mL Fe_3_O_4_@ZIF-8 NPs + 100 mT SMF). After 7 and 14 days of osteogenic induction, alkaline phosphatase (ALP) staining (Figure 3A) and quantitative activity analysis (Figure 3B) were performed. At each time point, the NPs + SMF group displayed more distinct ALP staining compared to the other groups. Furthermore, the quantitative ALP activity of the NPs + SMF group was significantly higher than that of the other groups. After 21 days of osteogenic induction, matrix mineralization, as an indicator of late-stage osteogenic differentiation, was evaluated via Alizarin red S (ARS) staining of calcium nodules (Figure 3C) [52]. Compared with the other groups, the NPs + SMF group exhibited more distinct calcium nodules formation, and semi-quantitative measurements indicated significantly greater calcium deposition in this group (Figure 3D). To further evaluate the osteogenic activity, RT-qPCR and Western blot were used to assess the expression levels of osteogenic genes and proteins, respectively. RT-qPCR results (Figure 3E) showed that, compared to the control group, the expression of *Alp*, osteocalcin (*Ocn*), and runt-related transcription factor 2 (*Runx2*) in the NPs and NPs + SMF groups was significantly upregulated, while the expression levels of these genes in the NPs + SMF group were higher than those in the NPs group. Western blot analysis (Figure 3F–I) demonstrated that the expression levels of ALP, OCN, and RUNX2 proteins were significantly higher in the NPs + SMF group compared with those in the other groups. These findings indicated that 100 µg/mL Fe_3_O_4_@ZIF-8 NPs combined with 100 mT SMF stimulation effectively promoted the osteogenic differentiation of BMSCs.

Taken together, 100 µg/mL Fe_3_O_4_@ZIF-8 NPs alone exhibited potent biocompatibility and favorable osteogenic activity. In contrast, the combination of 100 µg/mL Fe_3_O_4_@ZIF-8 NPs and 100 mT SMF stimulation not only retained excellent biocompatibility but also demonstrated significantly enhanced osteogenic potential in vitro, covering both the early and late stages of osteogenic differentiation. The strong osteogenic differentiation capacity exhibited by Fe_3_O_4_@ZIF-8 NPs may be primarily ascribed to the Zn^2+^ released from their degradation and the mechanical stimulation induced by the local magnetic field of Fe_3_O_4_ NPs. Furthermore, SMF stimulation alone and in combination with Fe_3_O_4_ NPs could promote osteogenic differentiation and bone regeneration, which may explain why the NPs + SMF group exhibited a higher in vitro osteogenic differentiation capacity than the other groups.

### 3.4. In Vitro Antibacterial Effect of Fe_3_O_4_@ZIF-8 NPs

*Staphylococcus aureus* (*S. aureus*) and *Streptococcus pyogenes* (*S. pyogenes*) are the most common bacteria causing jaw osteomyelitis (OM), which often involves mixed bacterial infections [53]. Additionally, *Escherichia coli* (*E. coli*) ranks among the most important pathogenic bacteria in such mixed infections. Accordingly, the antibacterial properties of Fe_3_O_4_@ZIF-8 NPs were assessed using *S. aureus*, *S. pyogenes*, and *E. coli*, with all experimental groups subjected to 100 mT SMF stimulation in all antibacterial activity assays, consistent with the magnetic field application in previous osteogenic experiments.

**Figure 4:**
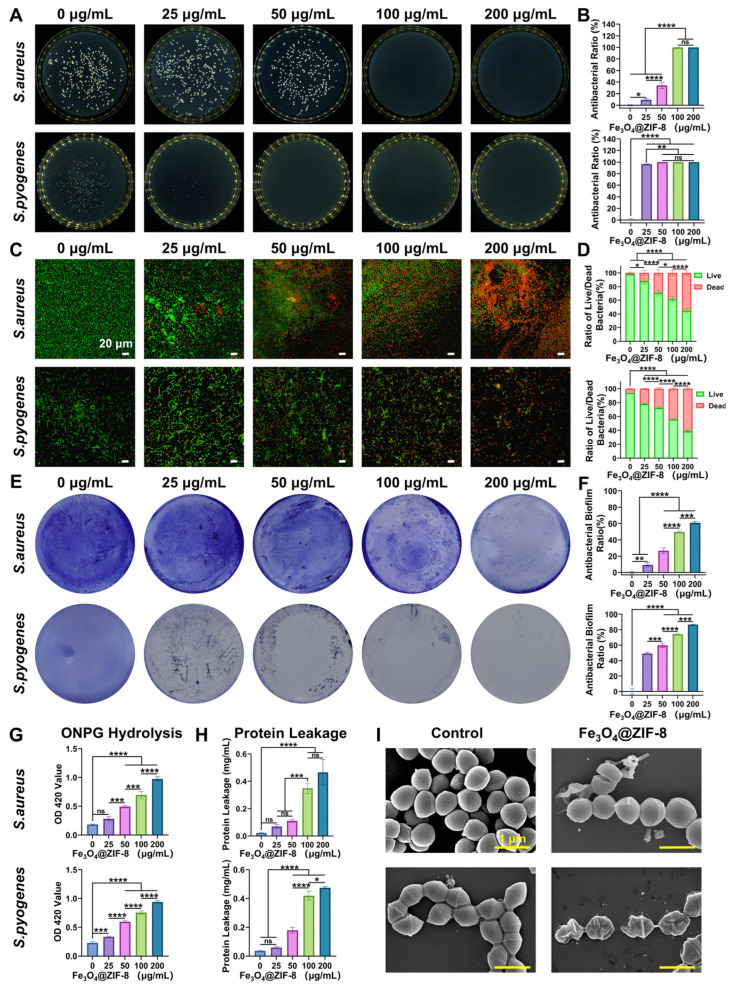
In vitro antibacterial effect of Fe_3_O_4_@ZIF-8 NPs. (**A**): Plate colony counting of *S. aureus* and *S. pyogenes*; (**B**): Quantitative analysis of bacterial colonies; (**C**): Live/dead staining fluorescence images of *S. aureus* and *S. pyogenes*. (**D**): Quantitative analysis of the live/dead bacterial ratio; (**E**): Crystal violet staining of *S. aureus* and *S. pyogenes* biofilms; (**F**): Quantitative analysis of antibacterial biofilm ratio; (**G**): Permeability of bacterial membrane of *S. aureus* and *S. pyogenes*; (**H**): Protein leakage of *S. aureus* and *S. pyogenes*; (**I**): SEM images of *S. aureus* and *S. pyogenes* after treatment without and with Fe_3_O_4_@ZIF-8 NPs. *n* = 3, ns: no significance, * *p* < 0.05, ** *p* < 0.01, *** *p* < 0.001, and **** *p* < 0.0001.

The minimum inhibitory concentration (MIC) of Fe_3_O_4_@ZIF-8 NPs against *S. aureus*, *S. pyogenes*, and *E. coli* was determined via resazurin staining (Figure S10A) [54]. Results demonstrated that Fe_3_O_4_@ZIF-8 NPs displayed concentration-dependent antibacterial activity, with MIC values of 100 µg/mL (*S. aureus*), 25 µg/mL (*S. pyogenes*), and 400 µg/mL (*E. coli*), respectively. This indicated better antibacterial activity against *S. aureus* and *S. pyogenes* than against *E. coli*, possibly due to differences in membrane structure between Gram-positive and Gram-negative bacteria [55]. The colony-forming unit (CFU) count is an important indicator for quantifying the antibacterial properties of antibacterial materials [56]. As shown in Figure 4A,B, Fe_3_O_4_@ZIF-8 NPs treatment led to a significant colony reduction on agar plates. Few or no colonies of *S. aureus* (≥100 µg/mL) or *S. pyogenes* (25 µg/mL) survived. At the same concentration, the reduction in *E. coli* colonies was relatively smaller (Figure S10B,C). Specifically, compared with the 0 µg/mL control group, the antibacterial rates of 100 µg/mL Fe_3_O_4_@ZIF-8 NPs against *S. aureus*, *S. pyogenes*, and *E. coli* were 99.84%, 99.99%, and 71.46%, respectively. Bacterial live/dead staining assay, which enables assessment of cell membrane integrity [57], further visualized antibacterial effects at different Fe_3_O_4_@ZIF-8 NPs concentrations [58]. All bacteria were stained with NucGreen (exhibiting green fluorescence), whereas dead bacterial cells were marked with EthD-III (displaying red fluorescence) (Figure 4C and Figure S10D). In the 0 µg/mL control group, minimal red fluorescence was observed for all bacterial species. In contrast, stronger red fluorescence was detected in the Fe_3_O_4_@ZIF-8 NPs-treated groups, with fluorescence intensity increasing with nanoparticle concentration, indicating a significant increase in bacterial mortality (Figure 4D and Figure S10E). These results indicated that Fe_3_O_4_@ZIF-8 NPs possessed excellent antibacterial properties and effectively disrupted the cell membrane integrity in a concentration-dependent manner.

Bacterial biofilms are three-dimensional structures formed when bacteria aggregate via extracellular matrices, and they are closely linked to chronic infections and antibiotic resistance [59]. To evaluate the inhibitory and disruptive effects of Fe_3_O_4_@ZIF-8 NPs on biofilm formation, crystal violet staining was used to visualize and quantify biofilms formed by *S. aureus*, *S. pyogenes*, and *E. coli* [60]. Among all groups, the 0 µg/mL control group exhibited the most extensive and darkest staining. In contrast, Fe_3_O_4_@ZIF-8 NPs treatment markedly inhibited biofilm formation and disrupted biofilms of all three species (Figure 4E and Figure S10F). Subsequently, the OD values of the crystal violet-stained biofilms were used to quantify the residual biofilm content. Compared with the 0 µg/mL control group, Fe_3_O_4_@ZIF-8 NPs treatment significantly reduced the biofilm biomass. Notably, at 100 µg/mL, the antibiofilm efficiencies against *S. aureus*, *S. pyogenes*, and *E. coli* were 49.55%, 74.18%, and 30.28%, respectively (Figure 4F and Figure S10G).

Understanding the precise antibacterial mechanisms is crucial for developing clinically applicable nanoantibiotics and antibacterial products [61]. Our results showed that Fe_3_O_4_@ZIF-8 NPs exhibited stronger inhibitory effects on *S. aureus* and *S. pyogenes* than those on *E. coli*, which may be attributed to variations in membrane charge among these bacterial strains. To further evaluate the effect of Fe_3_O_4_@ZIF-8 NPs on bacterial cell membranes, *o*-Nitrophenyl *β*-D-galactopyranoside (ONPG) was employed to assess membrane permeability [62]. When membrane permeability changes, ONPG readily penetrates bacteria, forming yellow *o*-nitrophenol. Thus, the membrane permeability can be quantified using this product. Figure 4G and Figure S10H showed that the membrane permeability of *S. aureus*, *S. pyogenes*, and *E. coli* increased significantly after Fe_3_O_4_@ZIF-8 NPs treatment, with increasing concentration. Typically, bacterial membrane disruption is accompanied by leakage of intracellular proteins [63]. Therefore, leaked proteins were quantified using a bicinchoninic acid (BCA) protein assay to reflect changes in bacterial membrane permeability (Figure 4H and Figure S10I). Compared to the 0 µg/mL control group, protein leakage in the Fe_3_O_4_@ZIF-8 NPs-treated groups was significantly higher, further confirming that Fe_3_O_4_@ZIF-8 NPs enhanced the membrane permeability. Subsequently, the morphological changes in the cell membranes of Fe_3_O_4_@ZIF-8 NPs-treated *S. aureus*, *S. pyogenes*, and *E. coli* were evaluated using scanning electron microscopy (SEM) (Figure 4I and Figure S10J). In the control group, all three bacterial species exhibited intact, smooth cell membranes. In contrast, Fe_3_O_4_@ZIF-8 NPs-treated bacteria displayed abnormal morphological changes, including surface roughness, wrinkling, and membrane breaches across all three bacterial species. These results indicated that Fe_3_O_4_@ZIF-8 NPs exerted antibacterial activity against *S. aureus*, *S. pyogenes*, and *E. coli* by disrupting their cell membranes.

Based on these in vitro findings, the combination of 100 µg/mL Fe_3_O_4_@ZIF-8 NPs and 100 mT SMF stimulation not only possessed outstanding biocompatibility and osteogenic potential but also exhibited excellent antibacterial activity. Given these merits, this combination was selected for subsequent in vivo studies to evaluate its therapeutic efficacy for jaw OM.

Notably, Fe_3_O_4_@ZIF-8 NPs exhibit a relatively high MIC of 400 μg/mL against *E. coli*, primarily due to the lipopolysaccharide (LPS) outer membrane of Gram-negative bacteria, which acts as a physical barrier, limiting nanoparticle penetration and efficient Zn^2+^ delivery [55,63]. Based on the core antibacterial mechanisms of the nanoparticles and the referenced literature [14,35,56], several concise, evidence-based strategies can be employed to enhance their efficacy against *E. coli*: Firstly, leveraging ZIF-8’s porous structure to load antibacterial payloads such as aztreonam or melittin that synergize with Zn^2+^—as demonstrated by Wang et al. (2024) [56], who showed antibiotic-loaded nanocarriers enhanced *E. coli* inhibition via membrane disruption and metabolic suppression, and Li et al. (2025) [14] who reported antimicrobial peptide (AMP)-loaded ZIF-8 reduced *E. coli* MIC by three-fold—Fe_3_O_4_@ZIF-8’s pH-responsive degradation ensures the co-release of Zn^2+^ and payloads, while magnetic targeting further boosts local concentrations [29]. Secondly, activating Fe_3_O_4_’s magnetothermal effect (MTE): under an alternating magnetic field (AMF), Fe_3_O_4_ generates 42–45 °C hyperthermia, which increases *E. coli* outer membrane permeability [35]. Zhang et al. (2025) [35] confirmed that this effect enhances Zn^2+^ penetration, reducing the required nanoparticle concentration by 50%, and that MTE also accelerates Zn^2+^ release and directly inactivates bacteria, aligning with the nanoparticles’ inherent magnetic responsiveness [19,29]. Thirdly, integrating photothermal agents (PTAs) such as Au NPs by coating Fe_3_O_4_@ZIF-8 enables NIR-triggered hyperthermia; Fe_3_O_4_’s magnetic targeting localizes NIR energy, and the synergy between photothermal therapy (PTT) and Zn^2+^ has been validated for Gram-negative infections [35,56]. Zhang et al. (2025) showed ZIF-8/Au@Fe_3_O_4_ NPs exhibited four-fold higher efficacy against *E. coli* under NIR irradiation, via photothermal-induced membrane disruption and enhanced Zn^2+^ internalization [35]. Fourthly, modifying the nanoparticle surface with LPS-targeting ligands or cationic polymers to overcome the LPS barrier: Li et al. (2025) [14] reported polymyxin B nonapeptide (PMBN)-modified ZIF-8 enhanced *E. coli* binding, while Si et al. (2023) [21] showed cationic ZIF-8 improved membrane penetration, both synergizing with Zn^2+^ to enhance antibacterial efficacy. Finally, clarifying the application scope of Fe_3_O_4_@ZIF-8: the nanoparticles are primarily designed for the treatment of jaw OM, where Gram-positive bacteria account for >80% of clinical cases [53]. For mixed infections involving *E. coli*, the aforementioned strategies can optimize efficacy. Meanwhile, for pure E. coli infections, the optimized system (e.g., AMP-loaded NPs combined with MTE/PTT) serves as a targeted therapy, consistent with the principle of personalized clinical treatment. In conclusion, the high MIC against *E. coli* is addressable via literature-validated strategies: payload loading, magnetothermal/photothermal synergy, and surface modification. These retain the nanoparticles’ core advantages while overcoming the Gram-negative outer membrane barrier. They ensure scientific rigor and translational potential for *E. coli*-related jaw OM mixed infections.

### 3.5. In Vitro Antibacterial Mechanisms of Fe_3_O_4_@ZIF-8 NPs

**Figure 5:**
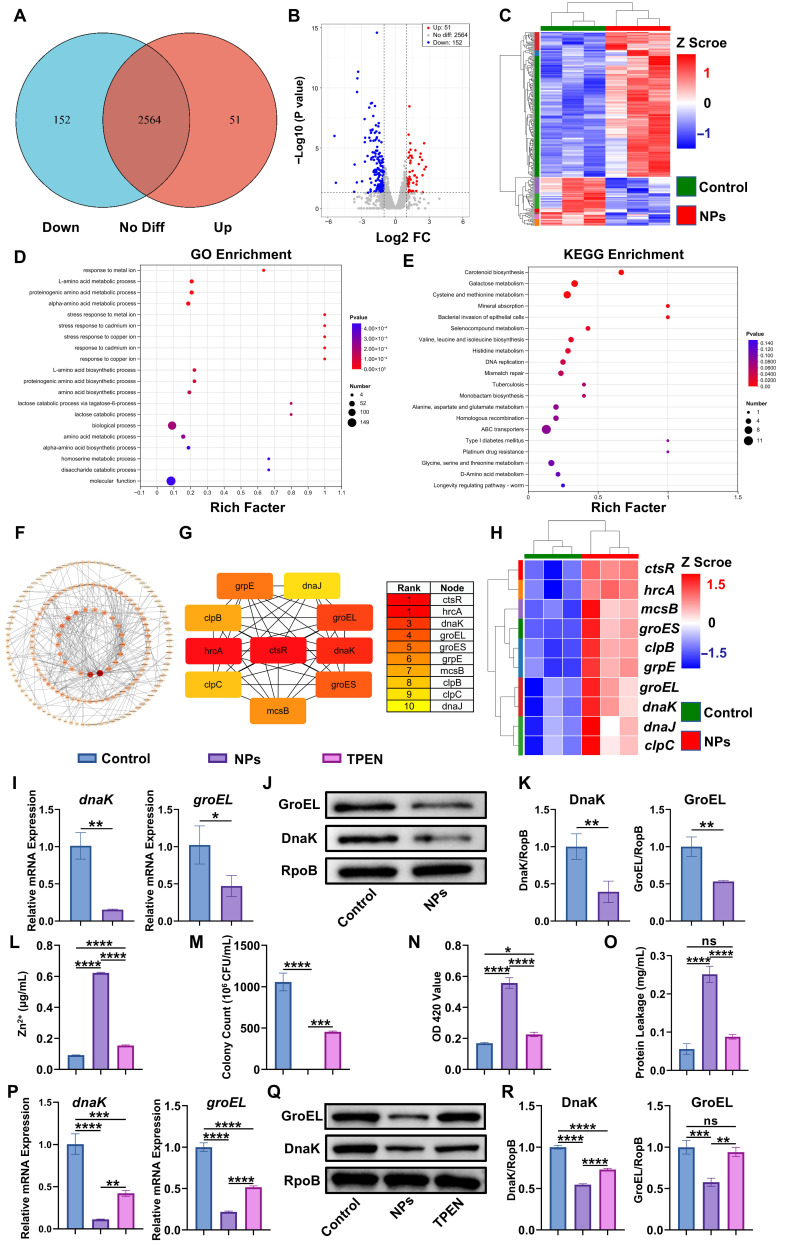
In vitro antibacterial mechanisms of Fe_3_O_4_@ZIF-8 NPs. (**A**): DEGs Venn diagram; (**B**): DEGs volcano plot; (**C**): The heatmap of all DEGs; (**D**): GO enrichment analysis of all DEGs; (**E**): KEGG enrichment analysis of all DEGs; (**F**): A PPI network of all DEGs; (**G**): The top 10 hub genes via the MCC algorithm; (**H**): The heatmap of the top 10 hub genes; (**I**): Relative expressions of *dnaK* and *groEL* mRNAs in *S. aureus* after different treatments; (**J**): Protein expressions of DnaK and GroEL; (**K**): Semi-quantitative analysis of protein expression; (**L**): Intracellular Zn^2+^ levels in *S. aureus* after different treatments; (**M**): Quantitative analysis of bacterial colonies; (**N**): Permeability of bacterial membrane of *S. aureus*; (**O**): Protein leakage of *S. aureus*; (**P**): Gene expression of *dnaK* and *groEL* in *S. aureus* after different treatments; (**Q**): Western blot of DnaK and GroEL proteins. (**R**): The Corresponding quantification of protein expression. *n* = 3, ns: no significance, * *p* < 0.05, ** *p* < 0.01, *** *p* < 0.001, and **** *p* < 0.0001.

Given that *S. aureus* is the most common bacterium in jaw OM and based on antibacterial susceptibility test results, *S. aureus* was selected as the bacterial model for transcriptomic sequence analysis. This analysis aimed to elucidate the precise molecular mechanisms mediating the antibacterial activity of Fe_3_O_4_@ZIF-8 NPs. The results of the RNA quality assessment showed that *S. aureus* RNA samples from both the control group (no NPs and SMF) and the NPs group (100 µg/mL Fe_3_O_4_@ZIF-8 NPs + 100 mT SMF) met the required quality standards for subsequent transcriptomic sequencing (Figure S11 and Table S4). An overview of the RNA sequencing results for the two groups is presented in Table S5. The raw transcriptomic datasets were deposited in the NCBI database (accession code: PRJNA1345057). Differentially expressed genes (DEGs) were analyzed based on the outcomes of transcriptomic comparisons. Collectively, 203 genes exhibited differential expression (with fold change > 2 and false discovery rate < 0.05), of which 51 were upregulated, and 152 were downregulated in the NPs group compared to the control group (Figure 5A,B). Furthermore, the heatmap highlighted the differences in all DEGs between the two groups (Figure 5C). To elucidate the potential association between DEGs and the antibacterial activity of Fe_3_O_4_@ZIF-8 NPs, enrichment analyses of Gene Ontology (GO) and Kyoto Encyclopedia of Genes and Genomes (KEGG) were performed. Specifically, GO enrichment analysis revealed that response to metal ion and stress response to metal ion were the two most significantly enriched biological processes (Figure 5D). Other major enriched biological processes included L-amino acid metabolic process, proteinogenic amino acid metabolic process, and alpha-amino acid metabolic process. Additionally, KEGG enrichment results indicated significant impacts on biosynthetic and metabolic pathways, particularly amino acid biosynthesis and metabolism, carotenoid biosynthesis, and galactose metabolism (Figure 5E). These GO-enriched biological processes and KEGG-enriched pathways may partially underpin the elucidation of the antibacterial mechanism of Fe_3_O_4_@ZIF-8 NPs.

To pinpoint the key DEGs, we constructed and analyzed a protein–protein interaction (PPI) network (Figure 5F) and identified the top 10 hub genes with the strongest connectivity and core functions using the maximum clique centrality (MCC) algorithm (Figure 5G). Notably, the top 10 hub genes, including *ctsR*, *hrcA*, *dnaK*, *dnaJ*, *grpE*, *groES*, *groEL*, *clpB*, *clpC*, and *mcsB*, are classic regulators or effectors of the heat shock response [64], implying that this response plays a key role in mediating the antibacterial activity of Fe_3_O_4_@ZIF-8 NPs against *S. aureus*. Transcriptomic sequencing results revealed that the expression of multiple heat shock response-related genes was significantly downregulated after Fe_3_O_4_@ZIF-8 NPs treatment (Figure 5H). As central components of the heat shock response, these genes are critical for *S. aureus* to mount adaptive responses against various stresses (e.g., temperature, oxidative, pH, and osmotic stress) [65]. At the core of this protective response is the production of large amounts of heat shock proteins (HSPs), a process tightly regulated by key transcriptional repressors, which are essential for bacterial survival [66]. In *S. aureus*, the heat shock response is primarily governed by two pivotal transcriptional repressors: HrcA and CtsR [67]. These repressors synergistically regulate distinct classes of heat shock genes to maintain stress adaptation and protein homeostasis. Specifically, HrcA regulates class I heat shock genes, including the canonical *dnaK* operon and *groESL* operon. The *dnaK* operon encodes the DnaK-DnaJ-GrpE chaperone system, with DnaK serving as the key member of the Hsp70 family [68]; and the *groESL* operon encodes the GroES-GroEL chaperone system, where GroEL acts as a central member of the Hsp60 family [69]. Notably, these two chaperone systems are indispensable not only for stress resistance but also for normal growth, remaining abundant across all metabolic states to support basal protein folding [64,66,68,69,70]. In contrast, CtsR regulates class III heat shock genes, primarily targeting the clp gene operons (e.g., *clpP*, *clpB*, and *clpC*), whose products (Clp proteases/chaperones) degrade misfolded proteins and enhance stress tolerance [71]. Under non-stress conditions, HrcA and CtsR bind to the promoters of their target genes to suppress transcription; upon stress stimulation, their inhibitory effects are relieved, allowing rapid induction of HSP synthesis [64].

Notably, the transcriptomic data further indicated that the expression of the encoding genes (*ctsR* and *hrcA*) for these two transcriptional repressors (CtsR and HrcA) and their respective target genes (*dnaK*, *dnaJ*, *grpE*, *groES*, *groEL*, *clpB*, *clpC*) were uniformly downregulated after Fe_3_O_4_@ZIF-8 NPs treatment. This suggested that Fe_3_O_4_@ZIF-8 NPs may disrupt the coordinated regulatory network of HrcA and CtsR, rather than simply interfering with the feedback regulation of individual repressors. To validate this, we performed RT-qPCR and Western blot to detect the expression of major heat shock response-related genes and proteins. The results of RT-qPCR confirmed that, compared to the control group, the transcriptional levels of *ctsR*, *hrcA*, *dnaK*, *dnaJ*, *grpE*, *groES*, *groEL*, *clpB*, *clpC*, and *mcsB* were significantly downregulated in the NPs group (Figure 5I and Figure S12), which was consistent with the transcriptomic data. Western blot results revealed that the protein expression levels of DnaK (Hsp70 family) and GroEL (Hsp60 family) were also significantly reduced in the NPs group (Figure 5J,K). Collectively, these results demonstrated that Fe_3_O_4_@ZIF-8 NPs treatment inhibited the core Hsp60 and Hsp70 chaperone families by disrupting the HrcA/CtsR-mediated heat shock regulatory network, thereby impairing the stress adaptation and survival capacity of *S. aureus*.

The coordinated downregulation of heat shock response-related genes in *S. aureus* can be explained by the unique characteristics of its heat shock regulatory network. First, because *hrcA* is nested in the *dnaK* operon, a tightly coupled nested regulatory network was formed [72]. Specifically, when CtsR represses the *dnaK* operon transcription, it may also directly repress *hrcA* transcription, thereby inhibiting HrcA synthesis. As an external factor, Fe_3_O_4_@ZIF-8 NPs may not only inhibit the transcription of the dnaK operon, but also interfere with the global transcriptional machinery required for *ctsR* transcription initiation and disrupt the negative autoregulation of CtsR. Second, GroEL supported HrcA function, and the DnaK-dependent ClpCP protease system regulated CtsR expression. Impairment of these chaperone/protease functions triggered a cascading downregulation of both repressors and their target genes, thus amplifying this effect via core chaperone dysfunction [73]. Third, since *hrcA*, *ctsR*, and their target genes in *S. aureus* all relied on the conserved bacterial transcription machinery for transcription initiation, the HrcA/CtsR network integrated with the global transcription system of *S. aureus* [64]. Fe_3_O_4_@ZIF-8 NPs may disrupt RNA polymerase activity or the interaction between sigma factors and heat shock gene promoters, thereby globally suppressing the transcription of HrcA/CtsR-controlled heat shock regulons.

To further investigate the relationship among Zn^2+^ released by Fe_3_O_4_@ZIF-8 NPs, cell membrane disruption, and the inhibition of heat shock response in *S. aureus*, N,N,N’,N’-Tetrakis (2-pyridinylmethyl)-1,2-ethanediamine (TPEN) was used to specifically chelate Zn^2+^ released by the nanoparticles [74]. *S. aureus* was divided into three groups: the control group (no NPs and SMF), the NPs group (100 µg/mL Fe_3_O_4_@ZIF-8 NPs + 100 mT SMF), and the TPEN group (100 µg/mL Fe_3_O_4_@ZIF-8 NPs + 100 mT SMF + 50 µM TPEN). Compared with the control group, intracellular Zn^2+^ levels in the NPs group increased significantly, whereas Zn^2+^ levels in the TPEN group decreased significantly (Figure 5L). The CFU counting assay showed that TPEN significantly abrogated the antibacterial efficacy of Fe_3_O_4_@ZIF-8 NPs, with a 42.96% increase in *S. aureus* survival (Figures S13 and Figure 5M). Moreover, TPEN co-treatment significantly decreased bacterial membrane permeability and the concentration of leaked proteins, indicating that Zn^2+^ plays a crucial role in disrupting bacterial cell membranes (Figure 5N,O). Notably, RT-qPCR (Figure 5P and Figure S14) and Western blot (Figure 5Q,R) results revealed that the expression levels of heat shock response-related genes and proteins were significantly upregulated after TPEN co-treatment, compared to the NPs group.

These findings, combined with antibacterial activity assays, membrane disruption assays, and transcriptomic analysis, clarify the sequential and hierarchical antibacterial mechanism of Fe_3_O_4_@ZIF-8 NPs, where Zn^2+^ release, membrane disruption, and heat shock response inhibition exert distinct yet synergistic effects in a strict causal sequence. Zn^2+^ serves as the initiating trigger, with the pH-responsive degradation of ZIF-8 in the acidic infectious microenvironment (pH~5.0) enabling rapid burst release of Zn^2+^ (5.48 µg/mL at 6 h). Meanwhile, only slow release occurs in neutral/alkaline environments (1.18 µg/mL at pH 7.4, 0.05 µg/mL at pH 10.0), ensuring targeted delivery to infected sites. As a divalent cation, Zn^2+^ then exerts immediate electrostatic interactions with the negatively charged bacterial cell membrane [21,63], disrupting the lipid bilayer within hours. This rapid physicochemical process accounts for ~30–40% of total antibacterial activity, causing cytoplasmic leakage and ionic homeostasis loss while serving as a necessary bridge for massive Zn^2+^ influx. This was directly visualized by SEM and quantified by ONPG hydrolysis and protein leakage assays. Intracellular Zn^2+^ overload, enabled by membrane disruption, subsequently targets the HrcA/CtsR-mediated heat shock response—the core stress resistance system that maintains bacterial protein homeostasis by upregulating heat shock proteins. Transcriptomic data showed the downregulation of 10 key heat shock response genes, RT-qPCR validated the reduced transcriptional levels of these genes, and Western blot verified reduced DnaK/GroEL expression, triggering irreversible collapse of protein homeostasis that eliminates bacterial stress adaptation and repair ability, accounting for ~60–70% of total antibacterial activity. TPEN chelation experiment directly confirmed this causality: chelating Zn^2+^ not only abrogated membrane disruption but also reversed the inhibition of heat shock response, verifying Zn^2+^ as the central effector and membrane disruption as a prerequisite for the inhibition of intracellular heat shock response.

Based on the above results, we can reasonably speculate that the specific antibacterial mechanism of Fe_3_O_4_@ZIF-8 NPs is that Zn^2+^ released by Fe_3_O_4_@ZIF-8 NPs directly disrupts the integrity of bacterial cell membranes and further causes disruption of bacterial ionic homeostasis, with the influx of high concentrations of Zn^2+^ being the most prominent. Subsequently, high concentrations of Zn^2+^ further mediate the inhibition of the heat shock response in *S. aureus*. This inhibitory effect triggers a comprehensive collapse of intracellular protein homeostasis, rendering bacteria unable to resist various adverse stresses and ultimately leading to bacterial death.

### 3.6. Characterization and Evaluation of an S. aureus-Infected Jaw Osteomyelitis Rat Model

*S. aureus* is the most widespread pathogenic microorganism associated with clinical jaw OM. To further verify the in vivo therapeutic effect of the combination of 100 µg/mL Fe_3_O_4_@ZIF-8 NPs and 100 mT SMF stimulation, we established a rat model of *S. aureus*-infected jaw OM and validated the model to ensure its stability [75]. For model induction, a 4 mm diameter defect was drilled on the left mandible of each rat (Figure S15). Subsequently, the model group was injected with 20 µL of 10^7^ CFU/mL *S. aureus* suspension into the defect, while the control group received an equal volume (20 µL) of phosphate-buffered saline (PBS) at the same site.

**Figure 6:**
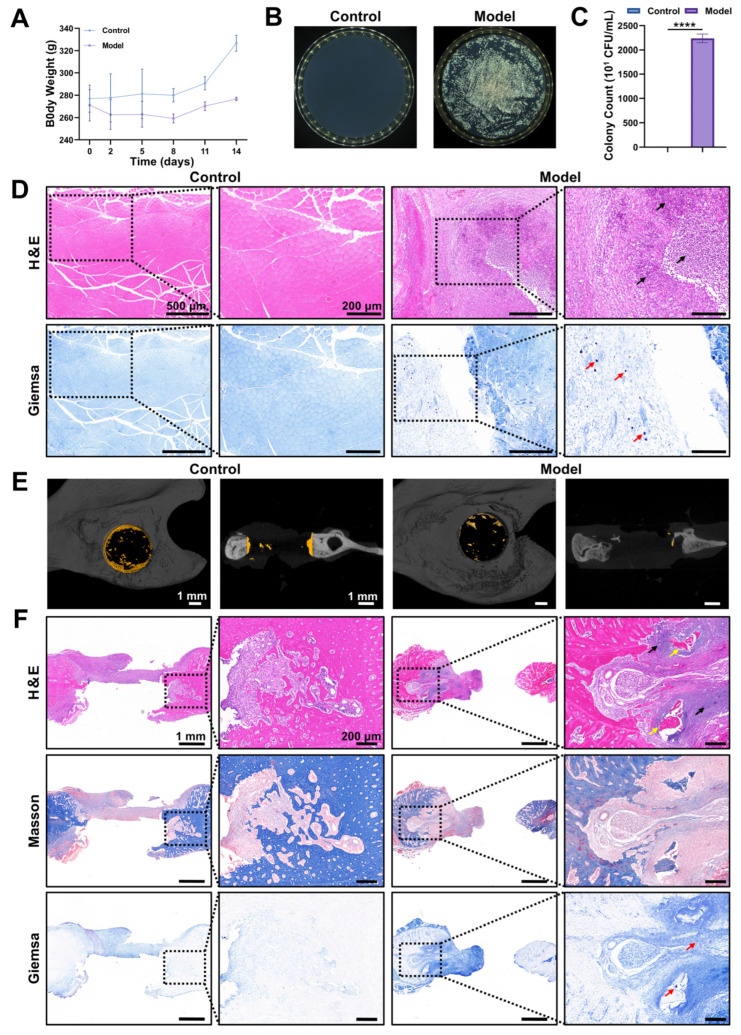
Establishment of an *S. aureus*-infected jaw osteomyelitis rat model. (**A**): Weight changes of rats in each group; (**B**): Plate colony counting of soft tissue; (**C**): Quantitative analysis of bacterial colonies; (**D**): H&E and Giemsa staining of soft tissue; (**E**): Micro-CT images and coronal cross-sections of mandibular tissues of rats in each group (yellow area: bone in a 4 mm diameter bone defect); (**F**): H&E, Masson, and Giemsa staining of the mandibular tissues of rats in each group (black arrow: inflammatory cells; red arrow: bacteria; yellow arrow: sequestra). *n* = 4, **** *p* < 0.0001.

At two weeks postoperatively, rats in the control group had higher body weight gain than those in the model group (Figure 6A). Compared with the control group, the model group showed proliferation of inflammatory granulation tissue and abscesses in the defect area, with pus appearing as a yellowish-white, viscous fluid (Figure S16). Furthermore, colony counts in the defect area (Figure 6B,C) showed that the bacterial load in the model group was significantly higher than in the control group. Additionally, Hematoxylin-eosin (H&E) and Giemsa staining (Figure 6D) of the soft tissues around the defect area revealed significant infiltration of inflammatory cells (emphasized by black arrows) and the presence of bacterial colonization (emphasized by red arrows) in the model group, whereas the control group showed almost none. Micro-computed tomography (micro-CT) images (Figure 6E) showed that, compared with the control group, the peri-defect cortical bone in the model group exhibited significant extensive bone resorption. Notably, no obvious new bone formation was detected in the model group, whereas only a few small bone fragments were observed, which were presumably free sequestra. Following decalcification of the mandible, H&E, Masson, and Giemsa staining (Figure 6F) were conducted to assess changes within the bone defect areas. Specifically, a modest quantity of newly formed bone matrix and collagen fibers was detected at the edges of the bone defect in the control group, with no obvious inflammatory cell infiltration. In contrast, the model group presented prominent abnormal pathological features marked by massive inflammatory cell infiltration (emphasized by black arrows) and bacterial colonization (emphasized by red arrows) at the defect edges, accompanied by bone resorption and free sequestra (emphasized by yellow arrows).

All of the above results indicated that the animals in the model group exhibited the general manifestations and histopathological characteristics of clinical jaw OM, demonstrating the successful establishment of the *S. aureus*-infected jaw OM rat model.

### 3.7. Therapeutic Effect of Fe_3_O_4_@ZIF-8 NPs on S. aureus-Infected Jaw Osteomyelitis

**Figure 7:**
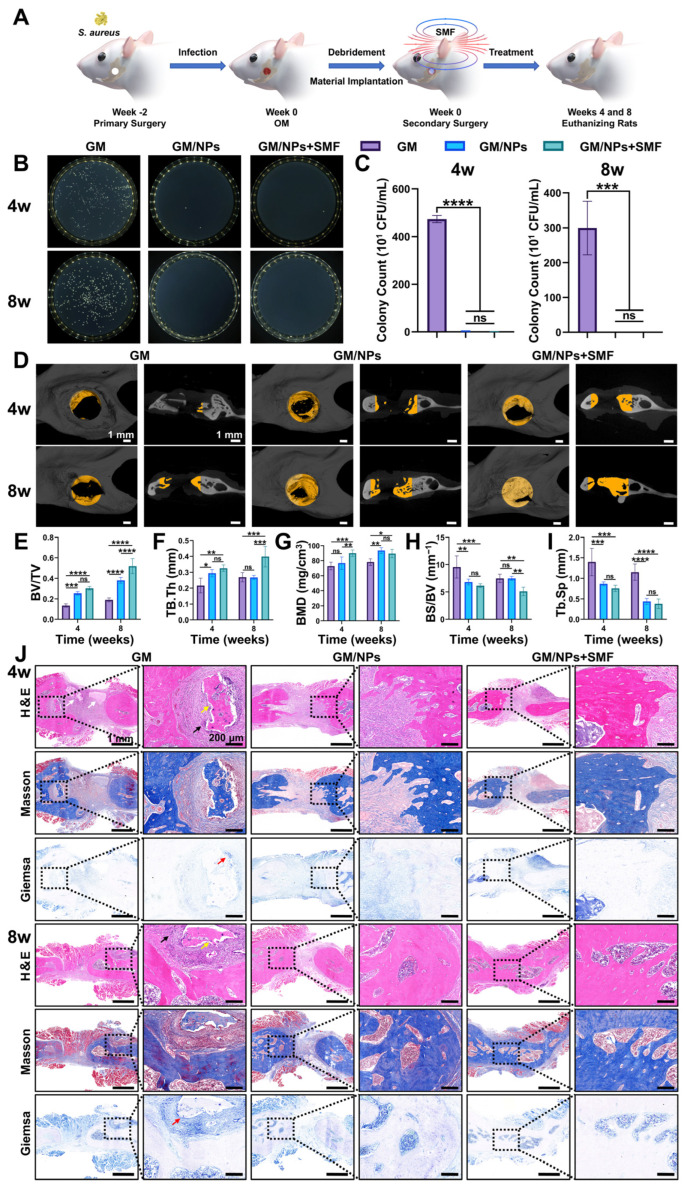
Therapeutic effect of Fe_3_O_4_@ZIF-8 NPs on *S. aureus*-infected jaw osteomyelitis. (**A**): Schematic illustration of establishing and treating an *S. aureus*-infected jaw osteomyelitis model; (**B**): Plate colony counting of soft tissue; (**C**): Quantitative analysis of bacterial colonies; (**D**): Micro-CT images and coronal cross-sections of mandibular tissues of rats in each group after 4 and 8 weeks of treatment (yellow area: bone in 4 mm diameter bone defect); (**E**–**I**): Quantitative analysis of micro-CT, including BV/TV, Tb.Th, BMD, BS/BV, and Tb.Sp; (**J**): H&E, Masson, and Giemsa staining of mandibular tissues of rats in each group (black arrow: inflammatory cells; red arrow: bacteria; yellow arrow: sequestra; white arrow: undegraded hydrogel). *n* = 4, ns: no significance, * *p* < 0.05, ** *p* < 0.01, *** *p* < 0.001, and **** *p* < 0.0001.

As illustrated in Figure 7A, to evaluate the in vivo antibacterial activity and bone regeneration capacity of Fe_3_O_4_@ZIF-8 NPs, all rats with *S. aureus*-infected jaw OM underwent debridement and were then randomly divided into three groups: the GM group (GelMA), the GM/NPs group (GelMA + 100 µg/mL Fe_3_O_4_@ZIF-8 NPs), and the GM/NPs + SMF group (GelMA + 100 µg/mL Fe_3_O_4_@ZIF-8 NPs + 100 mT SMF). The SMF setup used in the animal experiments is shown in Figure S17.

Compared with the GM group, CFU counting at the bone defect site (Figure 7B,C) showed a significant reduction in bacterial numbers in the GM/NPs and GM/NPs + SMF groups, indicating persistent infection in the GM group and effective mitigation of infection by Fe_3_O_4_@ZIF-8 NPs in the latter two groups. At 4 and 8 weeks after treatment, gross morphological observations of the harvested samples (Figure S18) revealed distinct differences. Specifically, the GM group exhibited overt signs of persistent infection at the bone defect site, with soft tissue appearing dark red to purplish (consistent with marked hyperemia), edematous and friable, and with poor bone defect healing. In contrast, the soft tissue at the defect site in the GM/NPs and GM/NPs + SMF groups appeared pale pink (closely resembling normal tissue color), firm, and well-vascularized, with reduced diameters or nearly healed defects. Micro-CT images (Figure 7D) showed that the GM/NPs + SMF group exhibited more new bone formation than the GM and GM/NPs groups. Specifically, at 4 weeks postoperatively, both the GM/NPs and GM/NPs + SMF groups exhibited evident new bone formation. By 8 weeks, the GM/NPs + SMF group had developed an almost complete mandibular structure, whereas the GM group still had substantial bone defects. To quantify new bone formation across groups, five key microstructural and mineralization parameters were analyzed: bone volume/tissue volume fraction (BV/TV), trabecular thickness (Tb.Th), bone mineral density (BMD), bone surface to tissue volume (BS/TV), and trabecular separation (Tb.Sp). Compared to the GM group, both the GM/NPs and GM/NPs + SMF groups exhibited significantly higher BV/TV ratios (4 and 8 weeks), Tb.Th values (4 weeks), and BMD values (8 weeks), and conversely lower BS/TV ratios (4 weeks) and Tb.Sp values (4 and 8 weeks). Notably, among the three groups, the GM/NPs + SMF group showed the highest BV/TV ratios, Tb.Th values, and BMD values, as well as conversely the lowest BS/TV ratios and Tb.Sp values (Figure 7E–I). Collectively, these findings demonstrated that the combination of 100 µg/mL Fe_3_O_4_@ZIF-8 NPs and 100 mT SMF stimulation effectively repaired bone defects in a rat model of *S. aureus*-infected jaw OM.

**Figure 8:**
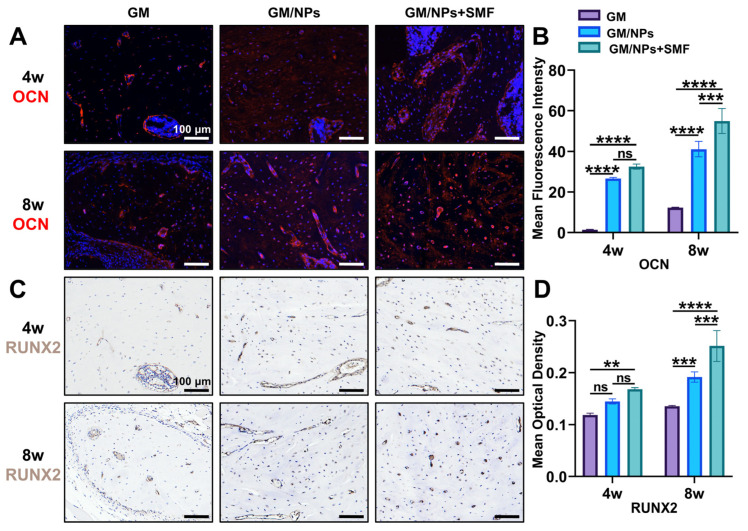
Histological evaluation of the mandibular tissues after 4 and 8 weeks of treatment. (**A**,**B**): OCN immunofluorescent staining and semi-quantification analysis (DAPI/OCN); (**C**,**D**): RUNX2 immunohistochemical staining and semi-quantification analysis. *n* = 4, ns: no significance, ** *p* < 0.01, *** *p* < 0.001, and **** *p* < 0.0001.

The therapeutic effects of the different treatment groups were further evaluated via H&E, Masson, and Giemsa staining (Figure 7J). At both time points, compared to the GM group, the GM/NPs and GM/NPs + SMF groups showed significantly fewer bacteria and inflammatory cells in bone defects, approaching near absence. Regarding bone regeneration, at 4 weeks post-treatment, the bone defect area in the GM group was filled with fibrous tissue heavily infiltrated by inflammatory cells (emphasized by black arrows) and bacterial colonization (emphasized by red arrows), accompanied by sequestra (emphasized by yellow arrows) and undegraded hydrogel (emphasized by white arrows). In contrast, the GM/NPs and GM/NPs + SMF groups showed prominent new bone formation. At 8 weeks post-treatment, most defects in the GM/NPs and GM/NPs + SMF groups were filled with mature, well-organized bone tissue. In contrast, the GM group exhibited persistent inflammatory cell infiltration, bacterial colonization, and free sequestra. These histological results demonstrated that 100 µg/mL Fe_3_O_4_@ZIF-8 NPs effectively eliminated infections and promoted mineralized new bone formation in *S. aureus*-infected jaw OM. Furthermore, 100 mT SMF stimulation accelerated the aforementioned bone-healing process, enhancing both the speed and quality of defect repair. The immunofluorescence (Figure 8A,B) and immunohistochemical staining (Figure 8C,D) results showed that the GM/NPs + SMF group exhibited a higher expression of OCN and RUNX2 in the bone defect area compared with the GM and GM/NPs groups. These findings were consistent with the results of RT-qPCR and Western blot in vitro osteogenic induction experiments. Moreover, H&E staining was conducted on heart, liver, spleen, lung, and kidney tissues (Figure S19). The results of this staining revealed that the interventions in all experimental groups failed to induce obvious damage or toxic effects on the structural integrity of the major organs of the rats. In summary, 100 µg/mL Fe_3_O_4_@ZIF-8 NPs effectively eradicated persistent infection and notably promoted bone formation in *S. aureus*-infected jaw OM, while 100 mT SMF stimulation further boosted and accelerated bone regeneration. Critically, the NPs + SMF combination also demonstrated favorable in vivo biosafety. Collectively, these merits provide a robust basis for subsequent clinical trials and biomedical translation of the treatment of jaw OM.

Despite the promising therapeutic efficacy of Fe_3_O_4_@ZIF-8 NPs combined with SMF in treating *S. aureus*-infected jaw OM, this study has several limitations that warrant consideration. First, a long-term toxicity assessment was not conducted. However, no obvious organ damage was observed at 8 weeks post-treatment. Nevertheless, the potential long-term biological effects of Fe_3_O_4_@ZIF-8 NPs remain to be elucidated. Second, biodistribution analysis of the nanoparticles was not performed, limiting understanding of their in vivo migration, targeting specificity, and clearance pathways. Third, the therapeutic effect was only validated in a rat model; larger animal models with more clinically relevant jawbone anatomy and physiological environments are needed to further confirm its translational potential. To address these limitations, future studies will focus on three key directions: conducting long-term biosafety evaluations to monitor organ function and nanoparticle accumulation; employing in vivo imaging techniques to track the biodistribution and clearance of Fe_3_O_4_@ZIF-8 NPs; and verifying the therapeutic efficacy in larger animal models to optimize dosage, administration routes, and SMF parameters. Additionally, further refinements of the nanoparticle formulation may improve clinical applicability, paving the way for the translation of this therapeutic strategy into clinical practice for jaw OM treatment.

## 4. Conclusions

In summary, Fe_3_O_4_@ZIF-8 NPs were rationally designed and successfully synthesized via a co-precipitation method for the treatment of jaw OM. Notably, the in vitro results demonstrated that 100 µg/mL Fe_3_O_4_@ZIF-8 NPs exhibited potent osteogenic activity, whereas their combination with 100 mT SMF stimulation further enhanced biocompatibility and osteogenic potential. In terms of antibacterial activity, Fe_3_O_4_@ZIF-8 NPs exhibited a favorable dose-dependent bactericidal effect. Furthermore, the underlying antibacterial molecular mechanisms of Fe_3_O_4_@ZIF-8 NPs were clarified. Specifically, Zn^2+^ released from Fe_3_O_4_@ZIF-8 NPs disrupted bacterial cell membrane integrity, following massive intracellular Zn^2+^ influx. High concentrations of Zn^2+^ inhibited the heat shock response, triggering a collapse of intracellular protein homeostasis that rendered bacteria unable to withstand adverse stresses and ultimately led to death. The in vivo results confirmed that 100 µg/mL Fe_3_O_4_@ZIF-8 NPs effectively eradicated persistent infections in an acidic infectious microenvironment and notably promoted bone formation in *S. aureus*-infected jaw OM, with 100 mT SMF stimulation further boosting and accelerating bone regeneration. Critically, the NPs + SMF combination also exhibited favorable in vivo biosafety. Collectively, the core–shell Fe_3_O_4_@ZIF-8 NPs platform, when combined with SMF stimulation, provides a promising therapeutic strategy to address the dual clinical challenges of antibiosis and osteogenesis in jaw OM.

## Data Availability

The original contributions presented in this study are included in the article/Supplementary Materials. Further inquiries can be directed to the corresponding authors.

## Supplementary Materials

The following supporting information can be downloaded at https://www.mdpi.com/article/10.3390/pharmaceutics18030359/s1.
