# Correction: Li et al. Multifunctional Fe_3_O_4_@ZIF-8 Nanoparticles with Antibiosis and Osteogenesis for Treatment of Jaw Osteomyelitis. *Pharmaceutics* 2026, *18*, 359

**DOI:** 10.3390/pharmaceutics18050623

**Published:** 2026-05-20

**Authors:** Heng Li, Zhiyue Zhang, Yu Wang, Ting Mou, Jiaqi Tian, Chong Huang, Lu Zhao, Zeyang Ge, Dandan Wang, Chenlu Li, Jihong Wang, Yanzhen Zheng, Lei Tian, Chunlin Zong

**Affiliations:** 1 State Key Laboratory of Oral & Maxillofacial Reconstruction and Regeneration, National Clinical Research Center for Oral Diseases, Shaanxi Clinical Research Center for Oral Diseases, Department of Oral and Maxillofacial Surgery, School of Stomatology, The Fourth Military Medical University, Xi’an 710032, China; 17798815711@163.com (H.L.); zhangzy126830@163.com (Z.Z.); mtmuting@126.com (T.M.); huangchongson@163.com (C.H.); zhaolujiayou0513@163.com (L.Z.); 18792863533@163.com (Z.G.); 17791823751@163.com (D.W.); lcl17309283208@outlook.com (C.L.); wangjh0129@163.com (J.W.); 2 Frontier Institute of Science and Technology, Interdisciplinary Research Center of Frontier Science and Technology, Xi’an Key Laboratory of Electronic Devices and Material Chemistry, School of Future Technology, Xi’an Jiaotong University, Xi’an 710054, China; 4121128016@stu.xjtu.edu.cn (Y.W.); zheng.yanzhen@xjtu.edu.cn (Y.Z.); 3 School of Stomatology, Jiamusi University, 258 Xuefu Street, Xiangyang District, Jiamusi 154007, China; 4 Xi’an Gaoxin No. 1 High School, No. 78 Xifengfu Road, Yanta District, Xi’an 710119, China; 13891949360@163.com

## Error in Figure

In the original publication [1], there was a mistake in Figure 3G,I as published. We wish to correct the error in the y-axis label of the quantitative analysis graphs for RUNX2 protein in Figure 3G,I. The original incorrect label was OCN/GAPDH, and it will be revised to the correct title RUNX2/GAPDH. The corrected Figure 3 appears below. The authors state that the scientific conclusions are unaffected. This correction was approved by the Academic Editor. The original publication has also been updated.

## Figures and Tables

**Figure 3:**
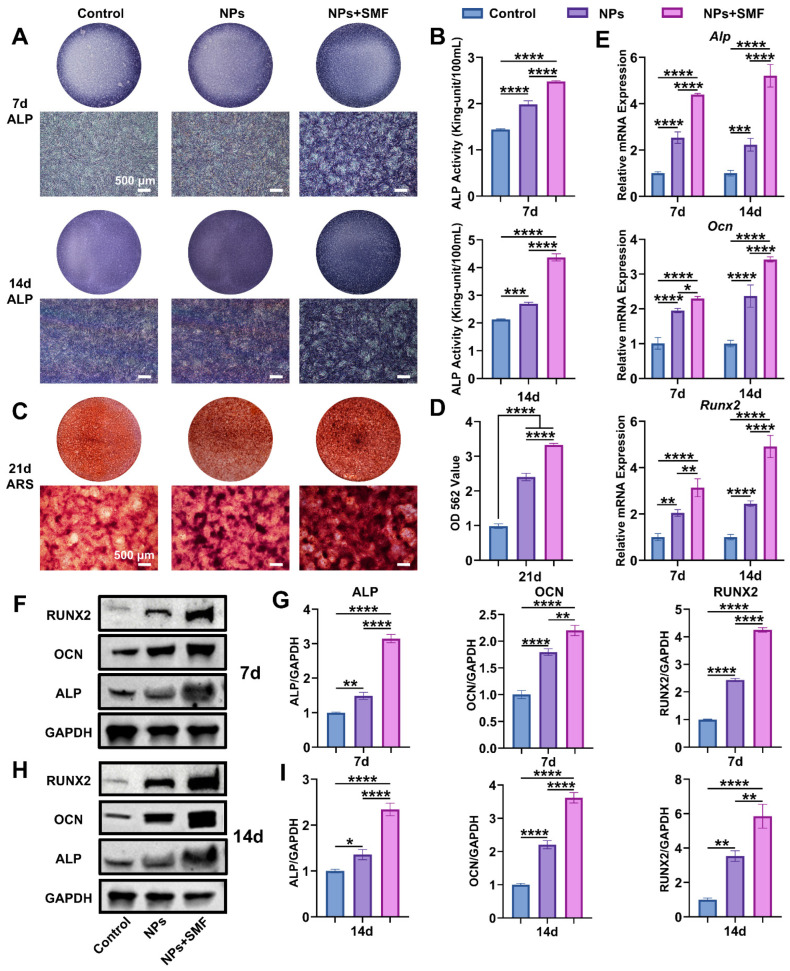
In vitro osteogenic effect of Fe_3_O_4_@ZIF-8 NPs. (**A**,**B**): ALP staining and quantification of ALP activity after osteogenic induction for 7 and 14 days; (**C**,**D**): ARS staining and semi-quantitative analysis after 21 days of osteogenic induction; (**E**): Relative mRNA expression levels of osteogenesis-related genes (*Alp*, *Ocn*, and *Runx2*) after induction for 7 and 14 days; (**F**–**I**): Western blot and quantification of osteogenesis-related proteins (ALP, OCN, and RUNX2) after induction for 7 (**F**,**G**) and 14 (**H**,**I**) days. *n* = 3, * *p* < 0.05, ** *p* < 0.01, *** *p* < 0.001, and **** *p* < 0.0001.
