# Supplementary material for: Multifunctional Fe3O4@ZIF-8 Nanoparticles with Antibiosis and Osteogenesis for Treatment of Jaw Osteomyelitis
Source: Pharmaceutics. 2026 Mar 13;18(3):359. doi: 10.3390/pharmaceutics18030359 (PMC13029378; doi:10.3390/pharmaceutics18030359)
Supplement: Supplementary file 1 [file pharmaceutics-18-00359-s001.zip › pharmaceutics-4160861-supplementary.pdf]

Supporting Information

Multifunctional Fe_3O_4@ZIF-8 Nanoparticles with Antibiosis and Osteogenesis for Treatment of Jaw Osteomyelitis

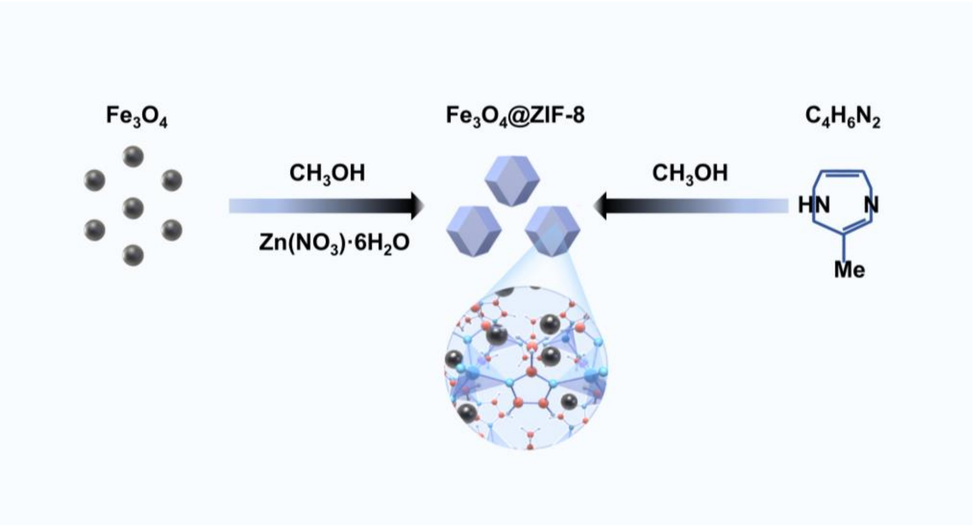

Figure S1 Schematic illustration of the synthesis process for Fe_3O_4@ZIF-8 NPs.

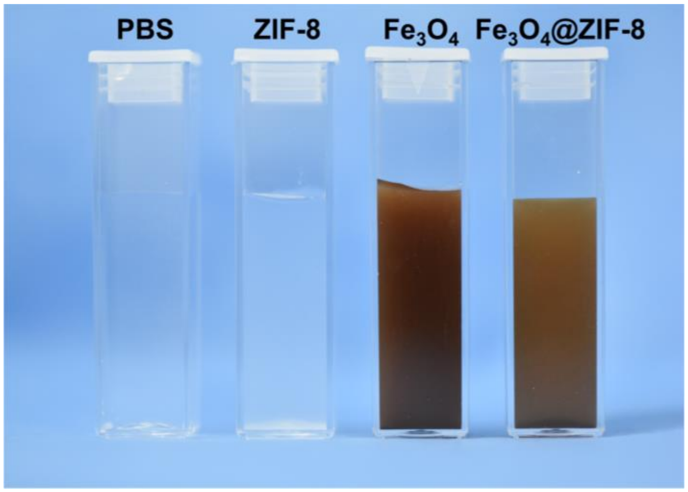

Figure S2 Visual appearance of PBS and aqueous suspensions of ZIF-8, Fe_3O_4, and Fe_3O_4@ZIF-8 NPs.

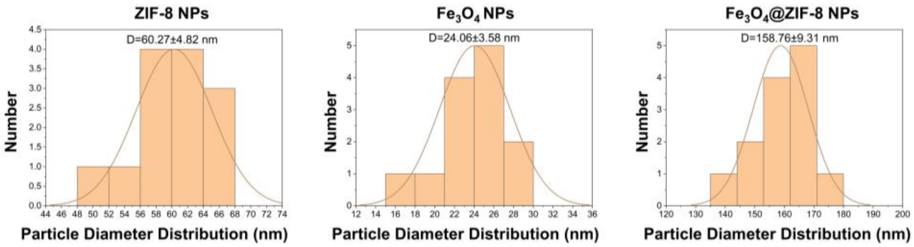

Figure S3 Particle diameter distribution of ZIF-8, Fe_3O_4, and Fe_3O_4@ZIF-8 NPs from TEM images.

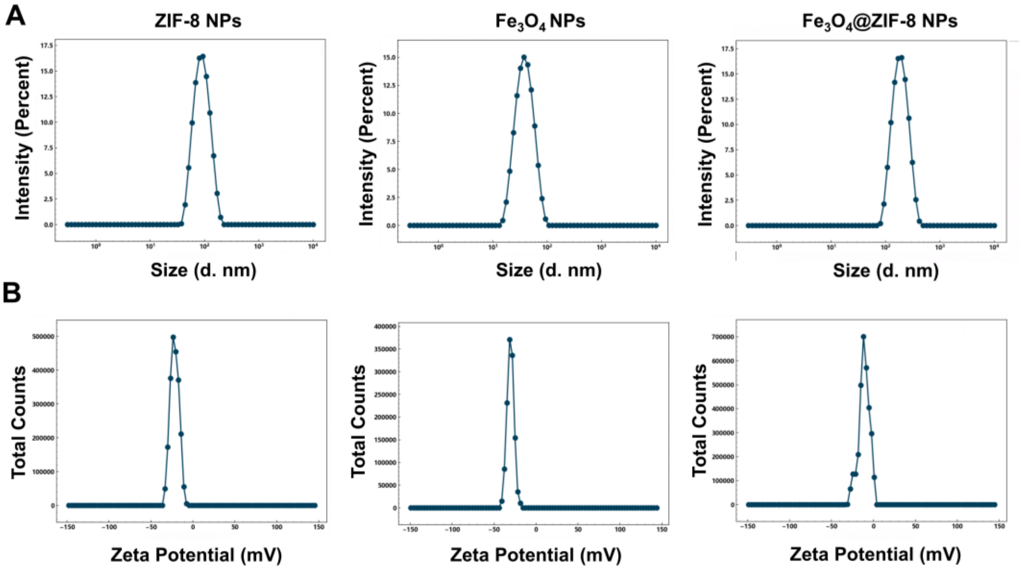

Figure S4 (**A**): Hydrodynamic size of Fe_3O_4@ZIF-8 NPs in PBS; (**B**): Zeta potentials of Fe_3O_4@ZIF-8 NPs in PBS.

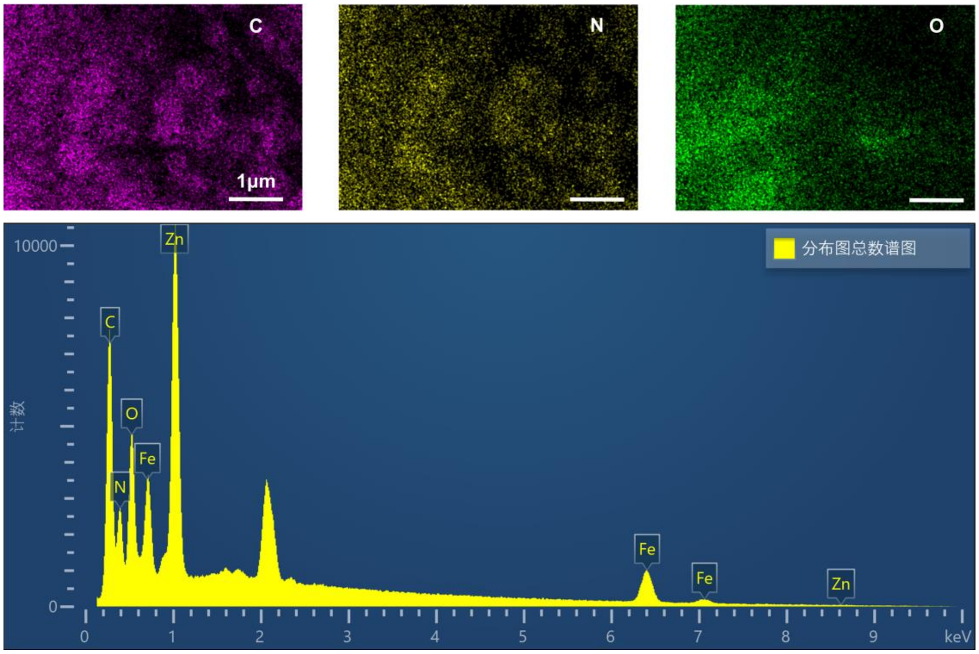

Figure S5 EDS mapping image, EDS spectrum of Fe_3O_4@ZIF-8 NPs.

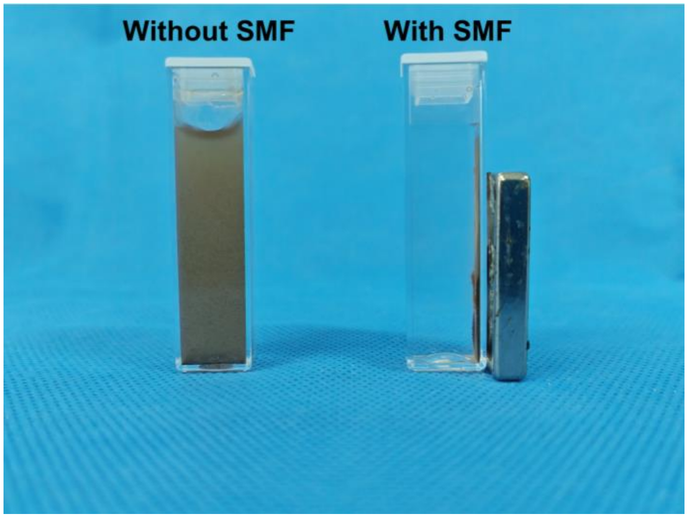

Figure S6 Photograph of aqueous suspensions of Fe_3O_4@ZIF-8 NPs without/with SMF.

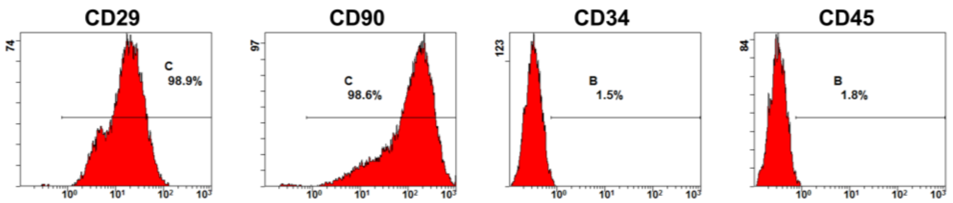

Figure S7 Flow cytometry identification of BMSCs specific surface markers (CD29, CD90, CD34, and CD45).

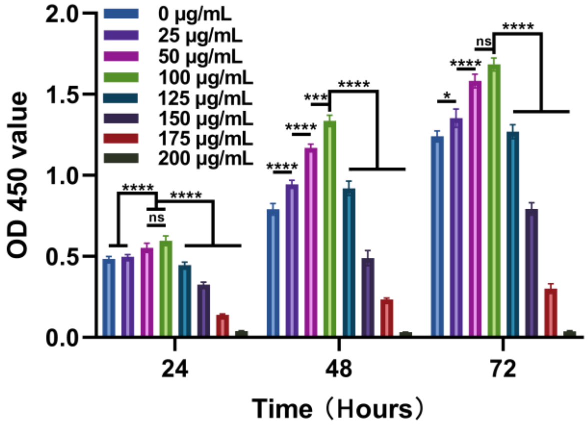

Figure S8 CCK-8 assay of BMSCs co-cultured with Fe_3O_4@ZIF-8 NPs at various concentrations (including 125, 150, and 175 μg/mL) for 24, 48, and 72 h. $n = 3$, ns: no significance, *$p < 0.05$, ***$p < 0.001$, and ****$p < 0.0001$.

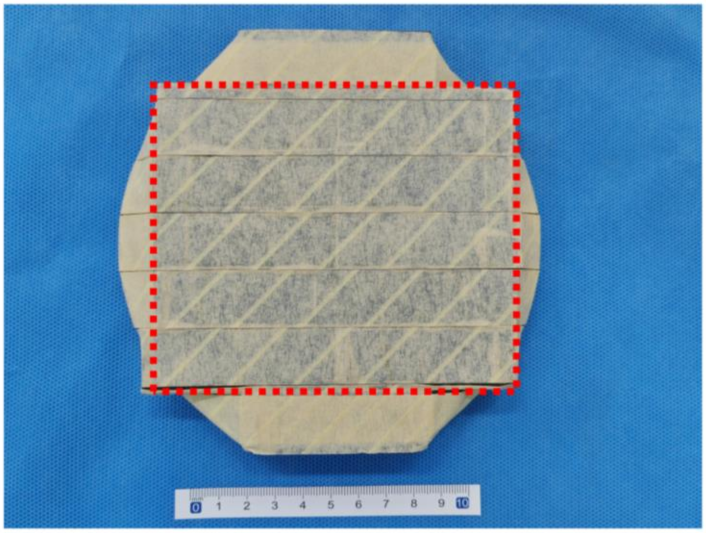

Figure S9 Photograph of the magnetic device for in vitro experiments.

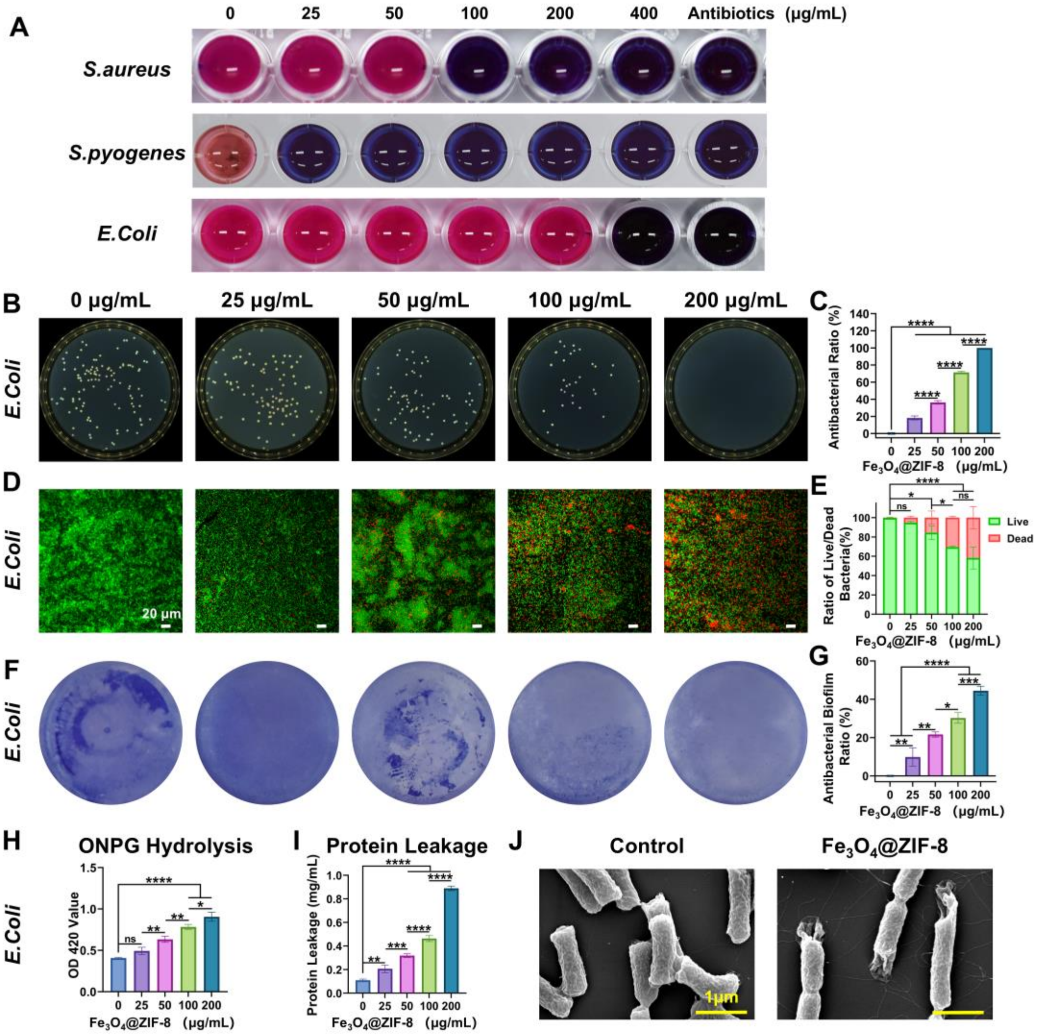

Figure S10 In vitro antibacterial effect of Fe_3O_4@ZIF-8 NPs. (**A**): Photograph of the MIC of Fe_3O_4@ZIF-8 NPs against *S. aureus*, *S. pyogenes* and *E. coli*; (**B**)： Plate colony counting of *E. coli*; (**C**)： Quantitative analysis of bacterial colonies; (**D**): Live/dead staining fluorescence images of *E. coli*; (**E**): Quantitative analysis of live/dead bacteria ratio; (**F**): Crystal violet staining image of bacterial biofilms of *E. coli*; (**G**): Quantitative analysis of antibacterial biofilm ratio; (**H**): Permeability of bacterial membrane of *E. coli*; (**I**): Protein leakage of *E. coli*; (**J**): SEM images of *E. coli* following treatment without or with Fe_3O_4@ZIF-8 NPs. n = 3, ns: no significance, *$p < 0.05$, **$p < 0.01$, ***p <

0.001, and ****$p < 0.0001$.

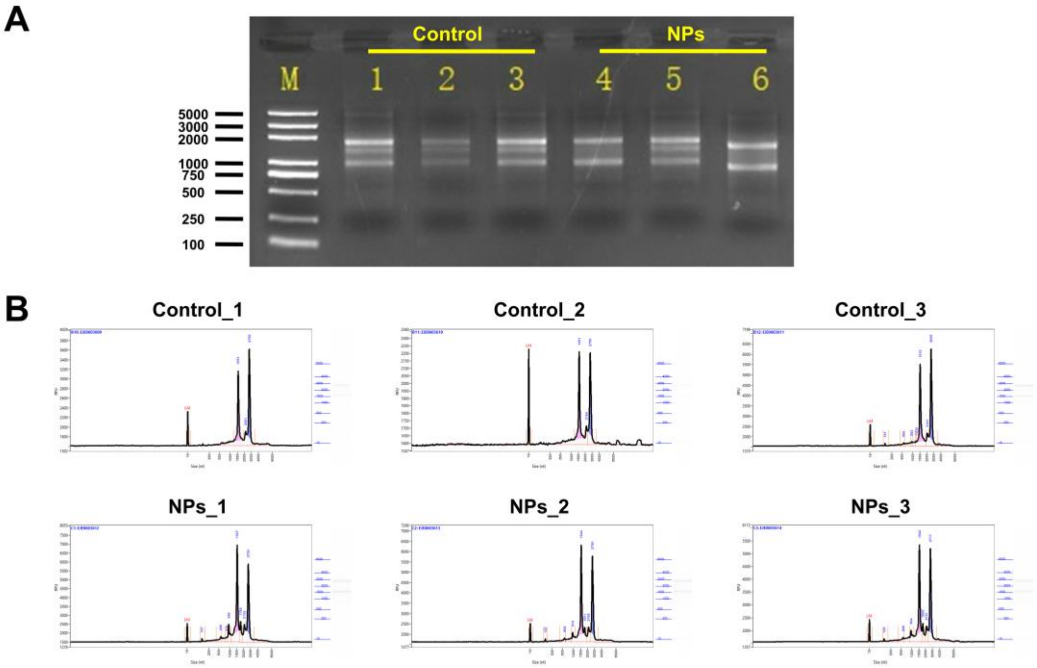

Figure S11 (**A**): RNA quality control of *S. aureus* in the control and NPs groups, confirmed by Aligent electropherograms. M: RNA lader; 1-3: the control group; 4-6: the NPs group; (**B**): rRNA ratio (23S/16S) and RNA quality number (RQN).

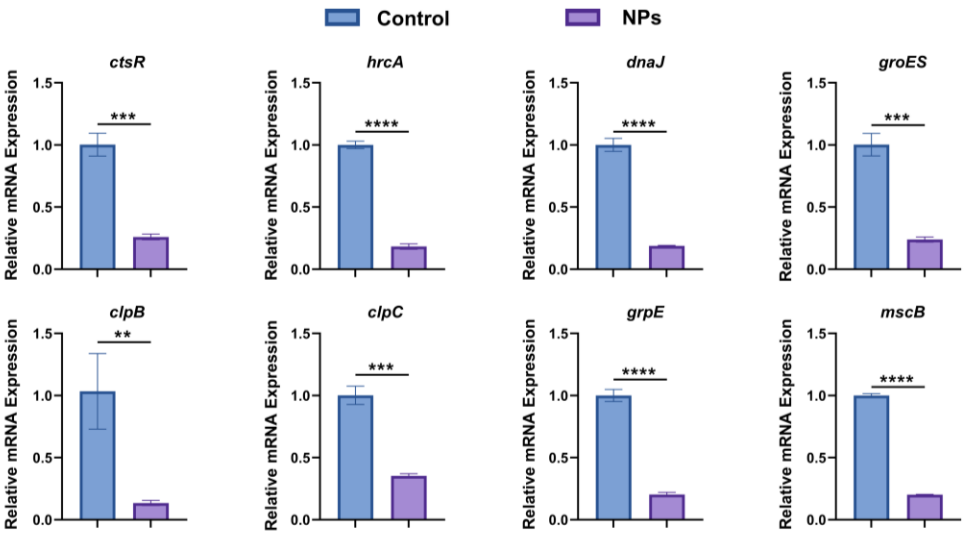

Figure S12 Gene expression of heat shock response-related genes in *S. aureus* with different treatments. n = 3, $^{**}p < 0.01$, $^{***}p < 0.001$, and $^{****}p < 0.0001$.

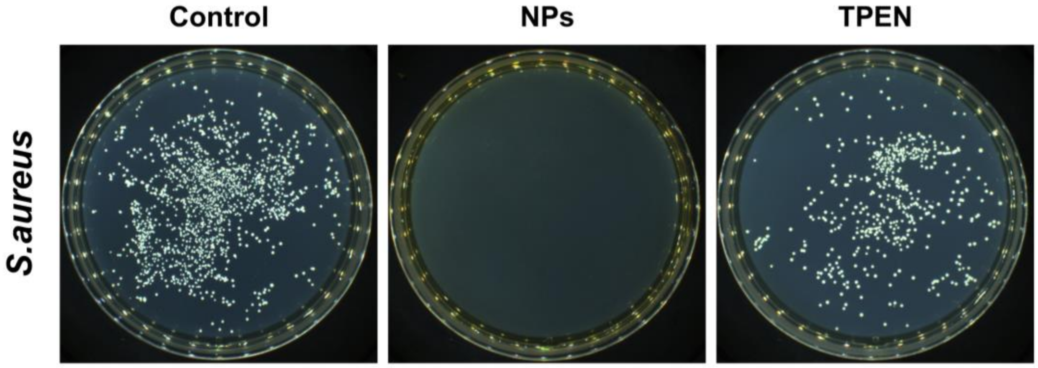

Figure S13 Photograph of plate colony counting for *S. aureus* with different treatments.

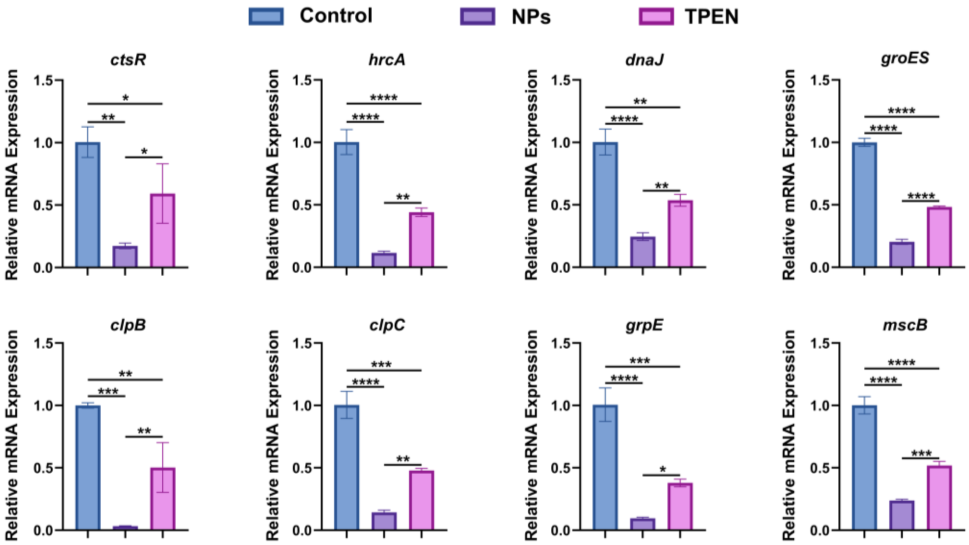

Figure S14 Relative expressions of heat shock response-related genes in *S. aureus* with different treatments. $n = 3$, *$p < 0.05$, **$p < 0.01$, ***$p < 0.001$, and ****$p < 0.0001$.

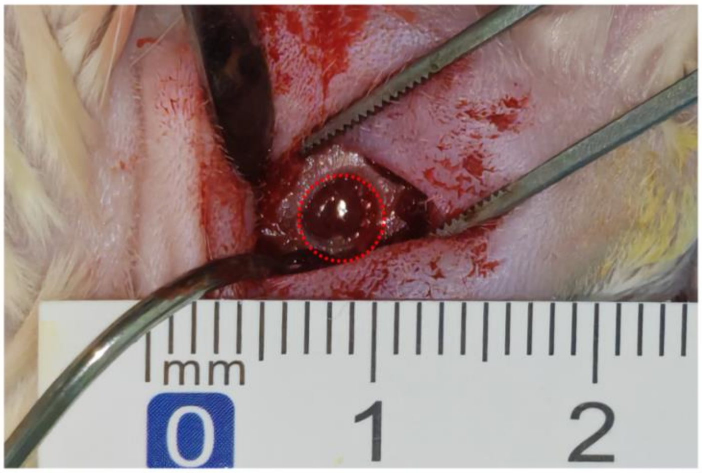

Figure S15 A 4-mm diameter defect on the left mandible of rat (red circle: initial size of bone defect).

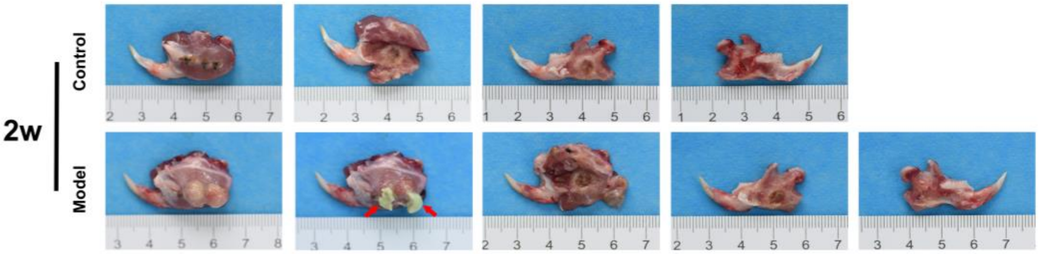

Figure S16 Gross morphology of left mandible and peripheral soft tissues of rats in control and model groups at 2 weeks post-treatment (red arrow: yellowish-white pus).

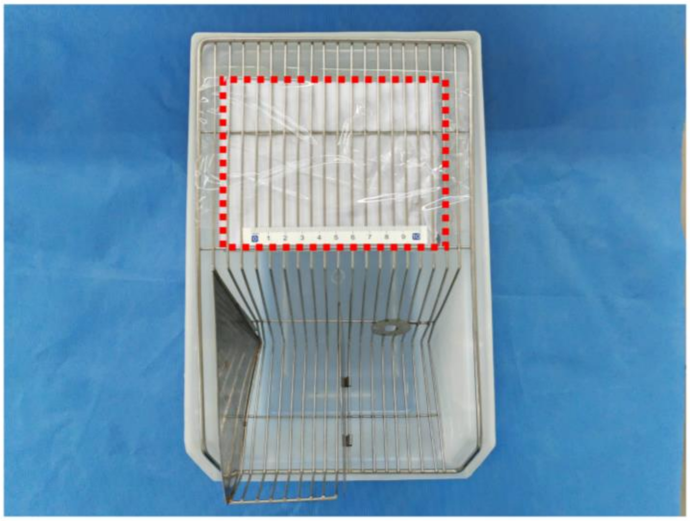

Figure S17 Photograph of the magnetic device for in vivo experiments.

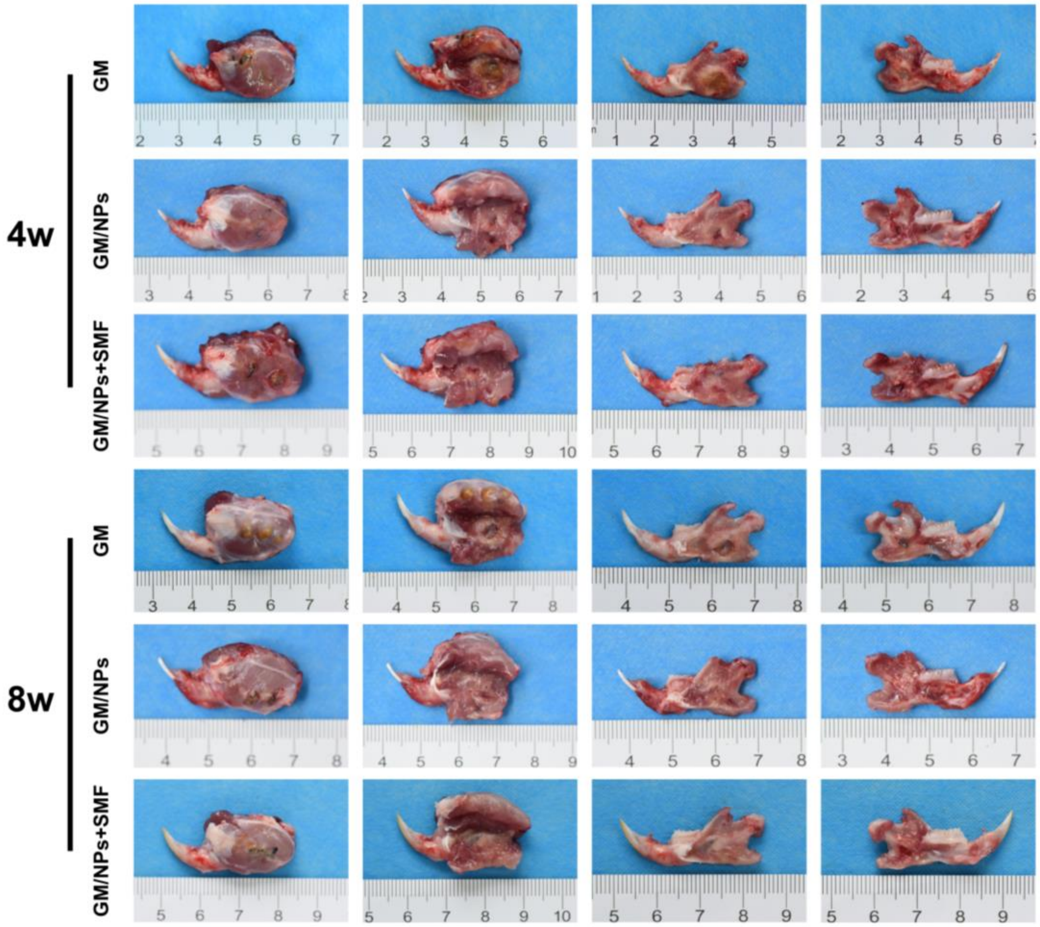

Figure S18 Gross morphology of left mandible and peripheral soft tissues of rats in each group after 4 and 8 weeks of treatment.

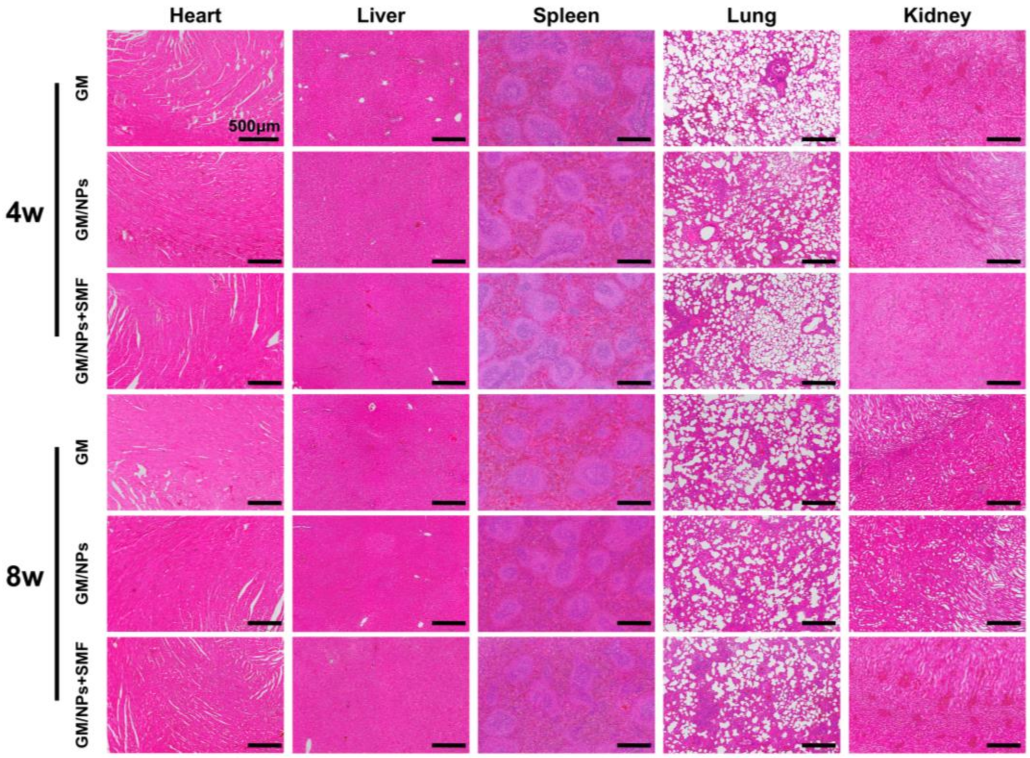

Figure S19 H&E staining of heart, liver, spleen, lung, and kidney tissues of rats in each group after 4 and 8 weeks of treatment.

Table S1 RT-qPCR primer sequences

Name	Primer	Sequence (5'–3')
Alp	Forward	ACTGGGGCCTGAGATACCC
Alp	Reverse	TCGTGTTGCACTGGTTAAAGC
Ocn	Forward	GGCGCTACCTGTATCAATGG
Ocn	Reverse	GTGGTCAGCCAACTCGTCA
Runx2	Forward	TGGTTACTGTCATGGCGGGTA
Runx2	Reverse	TCTCAGATCGTTGAACCTTGCTA
GAPDH	Forward	ACAACTTTGGTATCGTGGAAGG
GAPDH	Reverse	GCCATCACGCCACAGTTTC

Table S2 RT-qPCR primer sequences

Name	Primer	Sequence (5'–3')
ctsR	Forward	CACAGCGTTTTGATTGCGTACC
ctsR	Reverse	GATTCGGATGTAACCACCACCAC
hrcA	Forward	GCGACATAACTTGAATGTTAGTCCTG
hrcA	Reverse	CGAACGCCCTGAAGAACTATGTG
dnaK	Forward	CCTACACGCCAAGCAATGAAAGAC
dnaK	Reverse	CGGCATTAGAGCGTCGTTTCC
dnaJ	Forward	GTGGACAAGGATTCAATGGCTCTG
dnaJ	Reverse	TCTGCCGCCACCGAAGAAAG
grpE	Forward	AAACTGAAGGTGAAGCATTTGATCC
grpE	Reverse	GTTCTTGAGTGATTTCGCCAGATTC
groES	Forward	ACTGGACGCCTATTAAATGATGGTAC
groES	Reverse	CAGCATATTGTTGGAACACGACAC
groEL	Forward	TTTAACGCTGCTACAAACGAGTGG
groEL	Reverse	TGGAATTGATGCTACAACCGCTTC
clpB	Forward	CGGCATTAGAGCGTCGTTTCC
clpB	Reverse	ATTCAGCGGCAGCAACTAAGG
clpC	Forward	GCTTTATCTTCCTTGGACCAACTGG
clpC	Reverse	GTCTACACGGATCATCGCATCATC
mcsB	Forward	CAACCAGCAGCCGCAGTATTAG
mcsB	Reverse	GCCTGTAATGTCGTGTCAGTTCC
16S rRNA	Forward	GAGTACGACCGCAAGGTTGA
16S rRNA	Reverse	TGCACCACCTGTCACTTTGT

Table S3 EDS elemental quantitative analysis of Fe_3O_4@ZIF-8 NPs.

Element	Spectral Line Type	Wt%	Wt% Sigma	At%
C	K line type	31.58	0.26	52.97
N	K line type	13.40	0.27	19.28
O	K line type	10.11	0.12	12.73
Fe	L line type	22.59	0.34	8.15
Zn	L line type	22.32	0.18	6.88
Total Amount		100.00		100.00

Table S4 Summary for rRNA ratio (23S/16S) and RNA quality number (RQN) corresponding to Figure S11.

Majorbio Number	Sample Number	Gel Image Number	OD260/280	OD260/230	RQN
EJE0603609	Control_1	1	2.2	3.22	9.6
EJE0603610	Control_2	2	2.23	5.72	9.4
EJE0603611	Control_3	3	2.2	2.92	9.4
EJE0603612	NPs_1	4	2.2	2.76	9
EJE0603613	NPs_2	5	2.2	2.75	9.4
EJE0603614	NPs_3	6	2.18	2.78	9.2

Table S5 Summary for the RNA sequencing outcomes of the Control and NPs groups.

Sample Name	Raw reads	Clean Reads	Clean Bases (bp)	Clean Error Rate (%)	Clean Q20(%)	Clean Q30(%)
Control_1	20046708	19884880	2989086311	0.0118	99.29	96.15
Control_2	23126778	22971940	3457299976	0.0119	99.26	95.97
Control_3	22670140	22503810	3385429337	0.0118	99.29	96.27
NPs_1	21328028	21155464	3181349591	0.0117	99.34	96.44
NPs_2	19720564	19574918	2947327978	0.0116	99.36	96.51
NPs_3	22501848	22342528	3362413015	0.0119	99.24	95.89
